# Exploring mitochondrial diversity in the species-rich genus *Liolaemus*: the interplay between isolation by distance and environmental thermal variability

**DOI:** 10.1093/cz/zoaf075

**Published:** 2025-12-16

**Authors:** Mario R Ruiz-Monachesi, Félix B Cruz, Juan J Martínez

**Affiliations:** Laboratorio de Ecología Evolutiva y Biogeografía, INECOA- Instituto de Ecorregiones Andinas- CONICET- UNJu, Jujuy Y4600, Argentina; Instituto de Investigaciones en Biodiversidad y Medioambiente (CONICET—UNComahue) Bariloche, Río Negro R8400, Argentina; Laboratorio de Ecología Evolutiva y Biogeografía, INECOA- Instituto de Ecorregiones Andinas- CONICET- UNJu, Jujuy Y4600, Argentina

**Keywords:** body size, environmental heterogeneity, intraspecific variation, reproductive mode

## Abstract

Intraspecific genetic variation enhances a species´ capacity to endure diverse environments. Such variation can drive genetic divergence among populations, often manifesting as isolation by distance (IBD). Here, we investigated how ecological, environmental, life-history, body size, and phylogenetic factors shape IBD and nucleotide diversity (π) using two mitochondrial genes in *Liolaemus* lizards. From GenBank database, we examined two genes: *Cytochrome b* (*Cytb*; 88 species) and *12S ribosomal RNA* (*12S rRNA*; 37 species), integrating them with geographic information of each species. We then estimated Mantel's correlation coefficient (*r*), isolation-by-distance slope (β_IBD_), and π for each genetic marker, then evaluated the relationship between β_IBD_ and π. IBD was present in 55.40% of species for *Cytb* and 42.42% for *12S rRNA*. Species from higher altitudes were associated with steeper β_IBD_ in *Cytb*, whereas larger species showed a weaker β_IBD-*Cytb*_ effect. Bayesian generalized linear mixed models indicated that IBD positively explained π for both genes. In *Cytb*, π declined with increasing variability in environmental temperature and larger body size, whereas π in *12S rRNA* was negatively associated with annual mean temperature. Our results suggest that, although IBD and environmental temperature may generate shared patterns influencing mitochondrial genetic diversity in *Liolaemus* lizards, the two mitochondrial markers also exhibit distinct trends in how they shape genetic structure and diversity. This study represents an initial step toward understanding how mitochondrial genetic diversity may be maintained and structured within this species-rich genus.

## Graphical Abstract

**Graphical Abstract:**
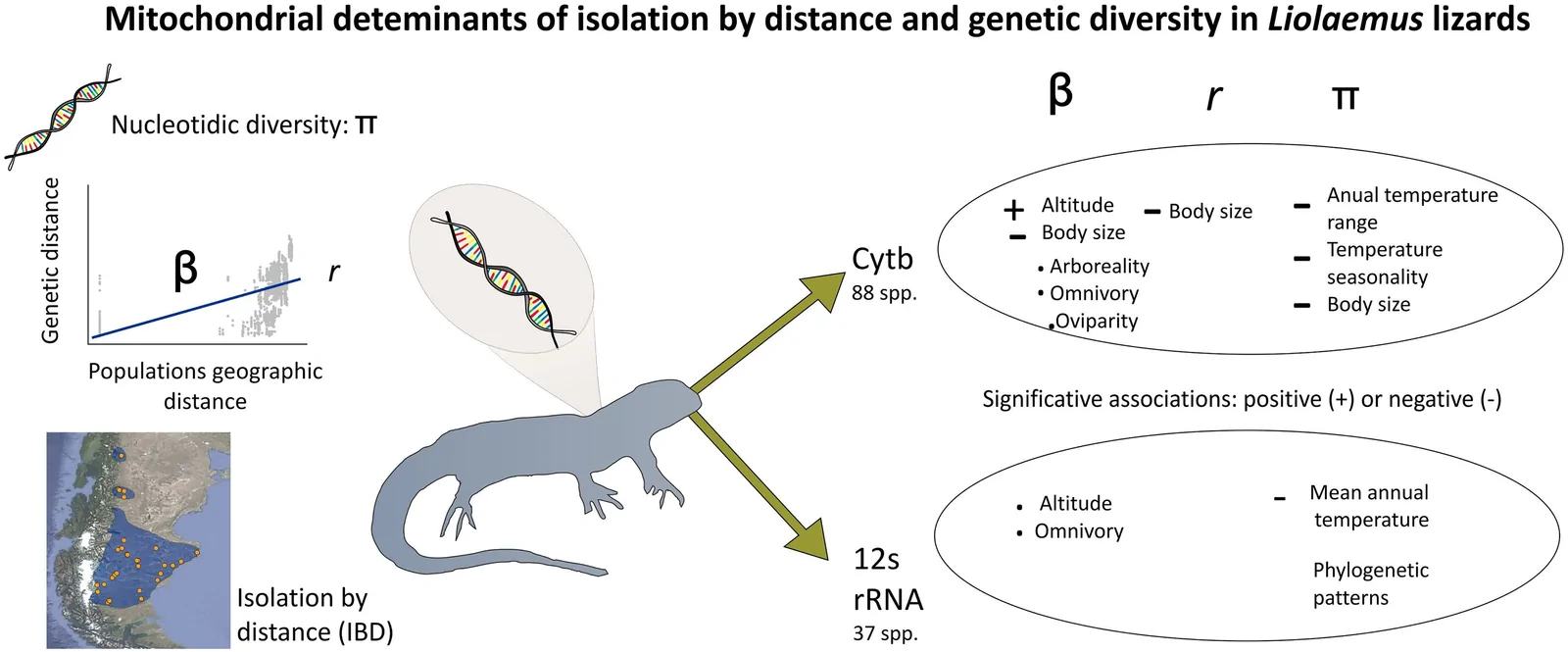
The graphic abstract of the study

Genetic variation within and among populations underpins the genetic structure of species (Frankham et al. 2002; Maya-Garcia et al. 2017). This variability is essential for persistence in heterogeneous and rapidly changing environments, as it enhances the adaptive potential (Frankham 1995; Jamieson and Allendorf 2012; Ekroth et al. 2019). Microevolutionary processes, including random mutation, genetic drift, and natural or sexual selection, generate and maintain such variation (Lande 1976; Lynch et al. 2016). Spatial structure further shapes diversity: when populations become geographically isolated, gene flow diminishes, potentially leading to genetic divergence and, in many cases, a decline in genetic similarity with increasing geographic distance (Wright 1943; Hancock and Hendrick 2018). Isolation by distance (IBD) is common in taxa with limited dispersal capabilities but may be weak or absent in highly vagile species (Hancock and Hendrick 2018). Numerous traits have been proposed as predictors of IBD strength, including ecological factors (diet, foraging mode; de Fraga et al. 2017), life-history traits (reproductive strategy, pace-of-life syndrome; Jenkins et al. 2010), taxonomy (He and Jiang 2014), ontogeny (Cayuela et al. 2020), and morphological characteristics (Dillon 1984). Body size is particularly relevant, as larger species often occupy broader ranges and may exhibit weaker IBD than smaller, range-restricted species (Singhal et al. 2022a).

Determining the drivers of intraspecific genetic diversity is equally important in conservation and evolutionary biology. Species at greater extinction risk generally exhibit low genetic diversity due to small, isolated populations prone to drift and inbreeding (Lande 1976; Hedrick and Kalinowski 2000; Frankham 2005; Rivers et al. 2014; O’Leary et al. 2015). Although larger effective population sizes (Ne) generally sustain higher genetic diversity, a range of intrinsic and extrinsic factors (including body size, life-history strategies, antipredator defenses, habitat characteristics), and broader environmental variables (such as area, climate, and latitude) have been associated with genetic diversity, although sometimes inconsistently (Ward et al. 1994; Romiguier et al. 2014; Ellegren and Galtier 2016; Smith et al. 2017; Brüniche-Olsen et al. 2018; Pelletier and Carstens 2018; López-Uribe et al. 2019; Mackintosh et al. 2019; Bolnick and Ballare 2020; Amador et al. 2024; Medina et al. 2024; Nava Martínez et al. 2024). Finally, phylogenetic history and marker type (coding vs. noncoding loci) can further influence genetic diversity (Barrow et al. 2021; Segovia-Ramiréz et al. 2023).

IBD and genetic diversity can be associated, but they represent distinct concepts. While IBD refers to the pattern whereby genetic similarity between populations decreases with increasing geographic distance, intraspecific genetic diversity quantifies the amount of genetic variation present within species. Spatial processes such as IBD may influence genetic diversity at the species level but are not its sole drivers. As a result, genetic diversity may vary independently of IBD within species, depending on factors such as mutation rates, historical connectivity, or habitat fragmentation (e.g., Segovia-Ramírez et al. 2023). For example, species with restricted gene flow across broad geographic gradients may retain high genetic diversity if mutation rates are elevated or if historical connectivity has been maintained (Vasconcelos et al. 2025). Conversely, low genetic diversity may arise in geographically isolated species, even without strong IBD signals, particularly under scenarios of habitat fragmentation or lineage-specific constraints (Pinto et al. 2024).

Reptiles, the third most threatened group of tetrapods, frequently exhibit IBD (Jenkins et al. 2010). Within them, different taxa can exhibit distinct patterns of IBD. For example, lizards generally show a stronger IBD effect than snakes (e.g., Singhal et al. 2022a); however, IBD does not necessarily lead to increased speciation rates in squamates (Singhal et al. 2022a). Studies on IBD and genetic diversity in lizards often focus on biogeography, niche specialization, behavior, or landscape genetics and have employed biogeographical and phylogenetic approaches (e.g., Howes et al. 2006; Hurston et al. 2009; Díaz-Cardenas et al. 2017; Reilly et al. 2023; Fenker et al. 2024; Fonseca et al. 2024; Keren-Rotem et al. 2024; Vasconcelos et al. 2025). Regarding the study of determinants of genetic diversity in reptiles, different factors have been proposed to explain this diversity, as follows: the number of museum occurrence records (a proxy for sampling effort), geographic position within a species' range and climatic/environmental factors (Singhal et al. 2017, 2022b; Grundler et al. 2019; Rivera et al. 2020), the geographic range size (Lau et al. 2025), or the size of settlements (Wall et al. 2024). Additionally, environmental heterogeneity, climatic, and ecological environmental factors can promote genetic diversity in reptiles (Rivera et al. 2020; Vasconcelos et al. 2025). Moreover, the relative importance of range size, reproductive mode, and phylogeny may vary depending on whether mitochondrial or nuclear genes are examined (Larkin et al. 2023).

With 290 species (Uetz et al. 2024) distributed from central Peru to Patagonia (Argentina and Chile) across a diverse range of environments (including forests, halophyte, rocky, sandy, and terrestrial habitats) and an elevation range spanning from sea level to 5,400 m (Cerdeña et al. 2021), the diverse and extensively studied genus *Liolaemus* (Abdala et al. 2021a, 2021b) serves as an ideal model for further exploration of these relationships. These lizards also exhibit variable habitat use; including arboreal specialists, sand dwellers, saxicolous species, and terrestrial generalists (Tulli et al 2011, 2012; Abdala et al. 2021a, 2021b). They also exhibit diverse diet types, ranging from herbivorous to insectivorous and omnivorous species (Espinoza et al. 2004; Ocampo et al. 2024). The genus *Liolaemus* is divided into two major phylogenetic clades: the Argentinean group (*Eulaemus*) and the Chilean group (*Liolaemus sensu stricto*) (Laurent 1983) and includes species with both oviparous and viviparous reproductive modes (Schulte et al. 2000; Esquerré et al. 2019). Viviparity, for instance, has been associated with high-elevation or cold-climate habitats (Schulte et al. 2000; Pincheira-Donoso et al. 2017; Esquerré et al. 2019; Cruz et al. 2022), suggesting that environmental pressures play a key role in shaping reproductive strategies. Similarly, ecological factors have also influenced dietary evolution within the genus. Herbivory has independently evolved multiple times (Espinoza et al. 2004), and herbivorous species tend to exhibit larger heads and distinct body shapes compared to other dietary groups (Ocampo et al. 2022), likely as adaptations to processing fibrous plant material. These evolutionary patterns highlight *Liolaemus*'s remarkable ecological diversity and adaptability across varied environments.

Previous studies for IBD patterns in *Liolaemus* lizards present opposite trends; some species, like *Liolaemus tenuis* and *L. lemniscatus*, exhibit clear IBD patterns (Vera-Escalona et al. 2012; Cab-Sulub and Álvarez-Castañeda 2022), while others, such as *L. wiegmanni*, do not (Villamil et al. 2017). Mixed results have also been reported in *L. bibronii* and *L. gracilis* (Morando et al. 2007). Genetic diversity studies in *Liolaemus* have primarily focused on taxonomic, phylogenetic, and phylogeographic aspects (Morando et al. 2007; Olave et al. 2011; Camargo et al. 2012; Grummer et al. 2018), with fewer studies emphasizing evolutionary and population genetics perspectives (e.g., Muñoz-Mendoza et al. 2017). Notably, no research has yet examined the association between IBD and body size or broad-scale determinants of genetic diversity in an evolutionary context for this genus.

In this study, we address three main objectives: (1) to test IBD and estimate the slope (β_IBD_) of genetic differentiation with geographic distance for each species, allowing us to assess potential determinants of the magnitude of IBD. Given that various ecological and biological factors may shape IBD patterns, we also test whether body size correlates with the presence of IBD, hypothesizing that larger species may exhibit weaker IBD patterns (Brüniche-Olsen et al. 2018). (2) To estimate species-specific nucleotide diversity (π) and identify potential drivers, including body size, life-history, ecological and climatic factors. Additionally, we assess the role of environmental heterogeneity by contrasting species with different reproductive modes (oviparous vs. viviparous) and phylogenetic groupings (*Eulaemus* vs. *Liolaemus sensu stricto*). (3) Investigate whether there is an association between β_IBD_ and nucleotide diversity (π). We hypothesize that species exhibiting stronger IBD patterns might also exhibit higher nucleotide diversity (Vasconcelos et al. 2025). By integrating these approaches, we assess whether variation in body size, life-history, ecological traits, phylogenetic relationships, and environmental conditions is associated with genetic diversity and spatial genetic structure in this species-rich genus.

## Materials and methods

### DNA sequence acquisition

We conducted a comprehensive search of the GenBank database (National Center for Biotechnology Information) up to 24 October 2023, to retrieve all available DNA sequences for *Liolaemus* lizards. For each sequence, we recorded the species name (or a tentative taxonomic assignment when identification was uncertain), GenBank accession and GI numbers, specimen identifiers, sequence length (bp), DNA molecule type, and associated publication details (authors, study title and journal) were recorded when available (details provided in Supplementary S1). Each referenced study was reviewed to assess data accuracy and reliability.

### Data filtration and preparation

Initial filtering excluded records that mentioned *Liolaemus* but lacked relevant genetic sequences. We focused on the most extensively studied markers, retaining only species with at least five sequences from different individuals to avoid pseudo replication and to ensure adequate representation of genetic variation within each species, following previous studies (e.g., Barrow et al. 2021). No geographic constraints were applied, as some species had specimens providing sequences from a single location (Supplementary S2). Taxonomic identities were verified using multiple systematic references (Supplementary S3). This process accounted for ongoing taxonomic revisions, ensured accurate species assignments, and addressed species complexes that had been split or renamed (e.g., *Liolaemus bibronii*, *L. elongatus*, *L. kingii*, *L. nigroviridis*, *L. wiegmanni*; see Supplementary S3). Only sequences linked to known geographic coordinates (Supplementary S2) were retained for further subsequent analyses. After evaluating various genetic markers (Fig. 1), we focused on *Cytb* and *12S rRNA*, as they were the most widely represented in the dataset (Fig. 1).

**Figure 1:**
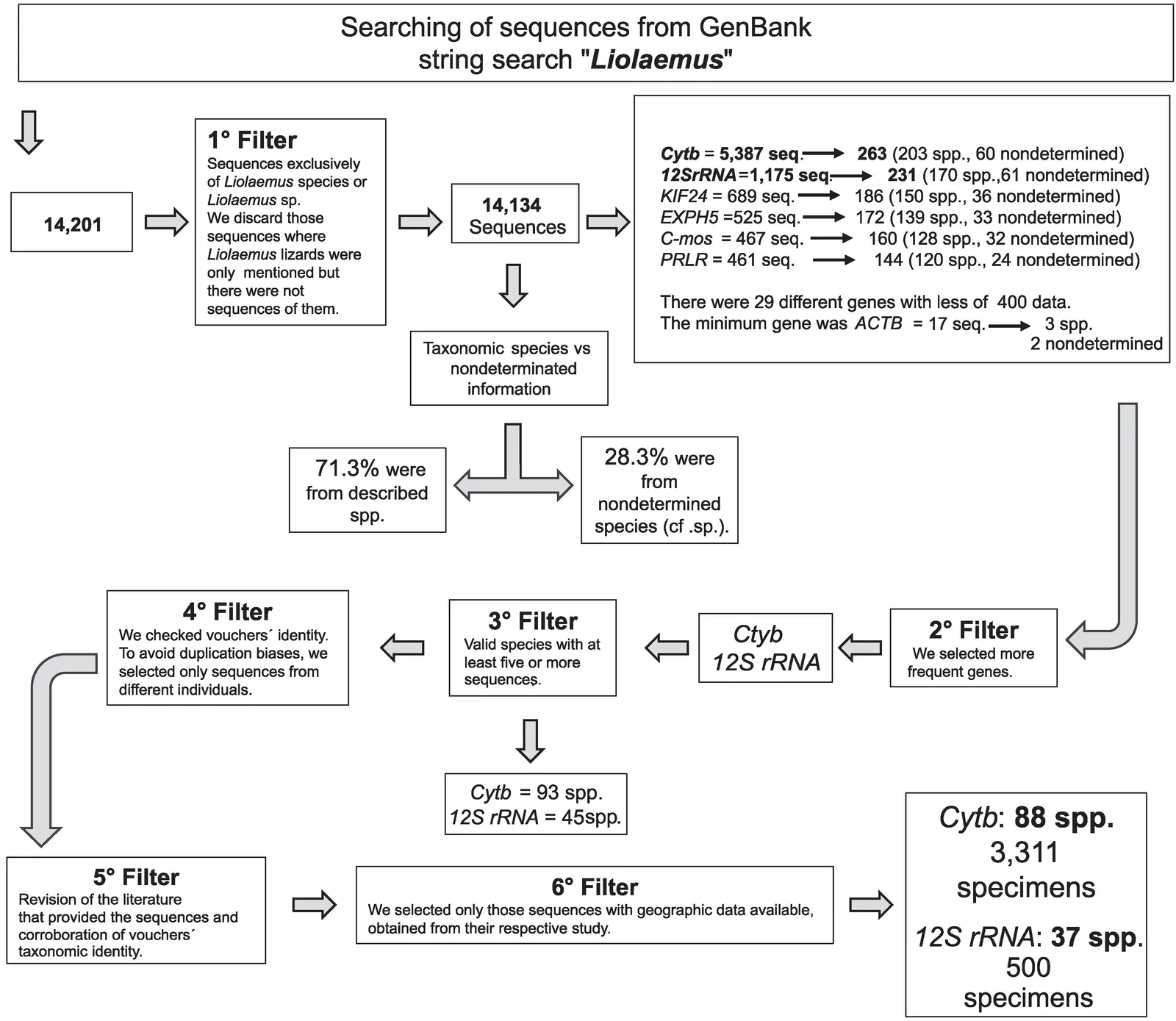
Flow diagram that illustrates the main steps used to assemble the final datasets of *Liolaemus* lizard sequences from the GenBank. Six successive filters were applied to the initial search results to ensure data accuracy and completeness, resulting in the final datasets for *Cytb* and *12S rRNA* analyses.

### Metrics of genetic variation

All analyses were performed in R version 4.3.3 (R Developed Core Team 2024). We downloaded sequences using the “read.GenBank,” saving them as FASTA files, with the “write.dna” (both functions from *ape* package; Paradis and Schliep 2019). Alignments were conducted for each species separately using MUSCLE v.3.8.31 (Edgar 2004) within SeaView5 v.5.0.5 (Supplementary S4) and subsequently edited in BioEdit sequence editor v.7.1 (Hall 1999). We recorded the final number of base pairs edited for each species (“bp,” Supplementary S4).

To calculate nucleotide diversity (π) per species, aligned-edited FASTA files were converted into DNA sequences format using the “readAAStringSet” (*msa* package; Bodenhofer et al. 2015) and “as.DNAbin” (*ape* package; Paradis and Schliep 2019) functions. We then calculated π using the “nuc.div” from the *pegas* package (Paradis 2010). This function sums the number of differences between pairs of sequences divided by the number of comparisons.

### Phylogenetic tree construction

Recognizing that species share a common phylogenetic history and should not be treated as independent statistical units (Felsenstein 1985), we performed phylogenetic corrections in our analyses. We constructed two metatrees: one for species with *Cytb* data and one for those with *12S rRNA*, using Mesquite v2.74 (Maddison and Maddison 2017). These trees integrated systematic information from multiple studies (Supplementary S3). Trees were ultrametrized by adjusting branch lengths according to Grafen's transformation (Grafen 1989) using “compute.brlen” method = “Grafen” (*ape* package; Paradis and Schliep 2019). Trees visualization was done with “plotTree” (*phytools* package; Revell 2024), resulting in the two phylogenetic frameworks used here (Fig. 2).

**Figure 2:**
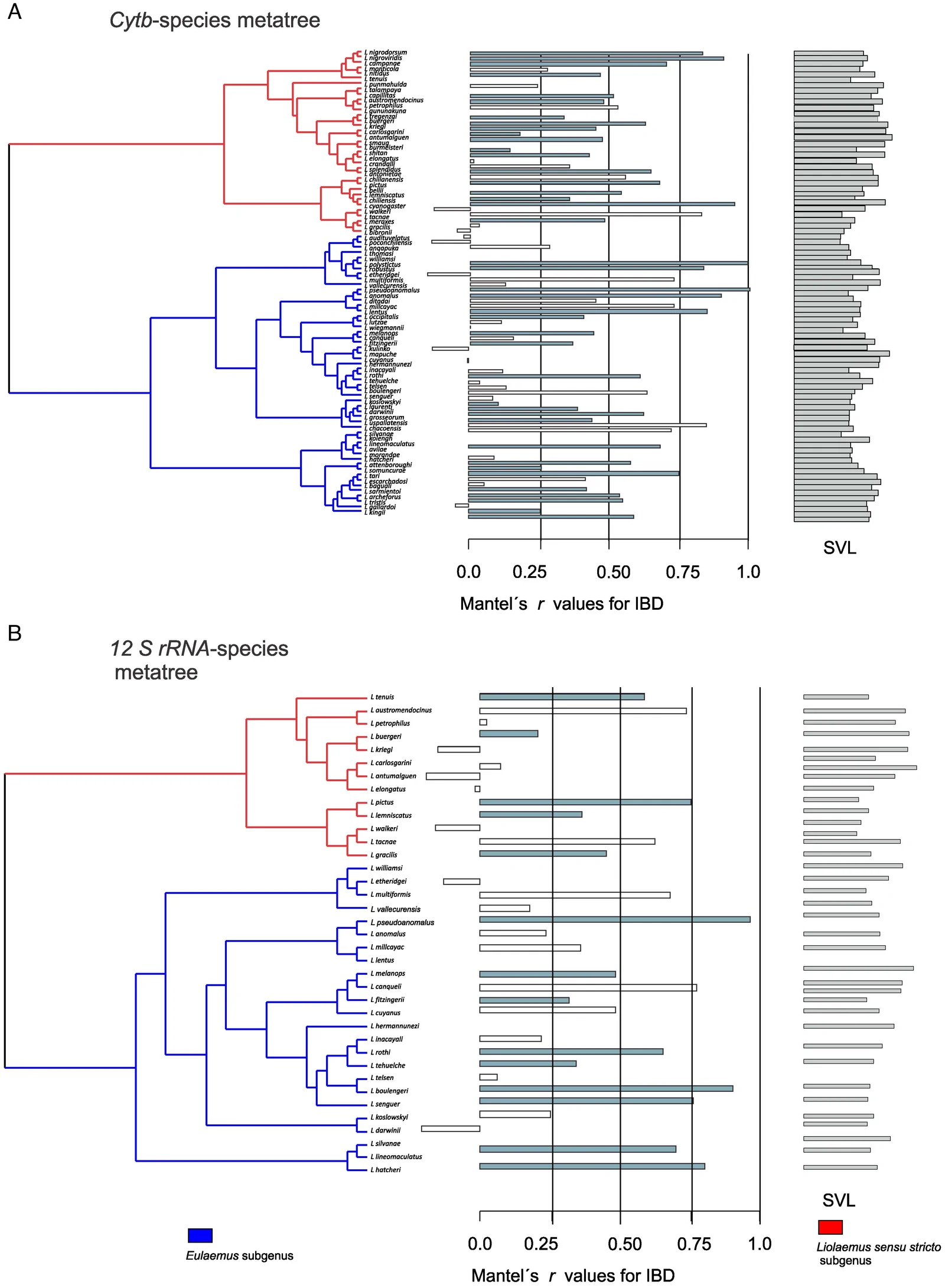
Meta-trees of *Liolaemus* lizards for (A) 88 species in the *Cytb* dataset and (B) 37 species in the *12S rRNA* dataset. Branch colours indicate subgenera: red for *Liolaemus sensu stricto* and blue for *Eulaemus*. The middle panels show the correlation coefficient (*r*) from Mantel test of isolation by distance (IBD). Coloured bars represent statistically significant *r* values, while white bars indicate non-significant *r* values. The right panels display each species' mean snout-vent length (SVL) (grey bars).

### Control variables

The final number of edited bp and the number of specimens (*n*) providing sequences for each species were used as control variables when necessary (Table 1).

**Table 1:** Variables used to examine potential determinants of genetic diversity in *Liolaemus* lizards.

Variable	Units of measurement	definition/levels
+bp		Final number of edited base pairs per species.
+n		Number of donor specimens used as vouchers for sequence extraction.
Diet type		Herbivorous, insectivorous, omnivorous
Identified threats		Yes, no
IUCN threatcategories		DD (data deficient), EN (endangered), LC (least concern), NE (not evaluated), NT (near threatened), VU (vulnerable)
SD		Sexual dimorphism; Absent, present
SU		Substrate use; Arboreal, halophyte, psammophilous, saxicolous, terrestrial
RM		Reproductive mode; Oviparous, viviparous
AREA	km^2^	Total area where species occur
SVL	mm	Average snout-vent length (body size) per species
***SVL max		Maximum snout-vent length per species
ALT	m	Altitude: elevation above sea level (masl) where each species occurs
BIO 1	°C	Annual mean temperature.
***BIO 2		Annual mean diurnal range: Mean of monthly temperature ranges (max temp—min temp)
*BIO 3		Isothermality: Indicates relative oscillation of day-to-night temperatures compared to annual oscillations.
*BIO 5		Maximum temperature of the warmest month: Highest monthly temperature in a year or averaged span of years
*BIO 6		Minimum temperature of the coldest month: Lowest monthly temperature in a year or averaged span of years
BIO 7		Annual temperature range: Variation in temperature over a period, examining the impact of extreme temperature conditions
*BIO 12	mm^3^	Annual precipitation: Total sum of monthly precipitation values
*RAD	W	Radiation values where each species occurs
**VAPR	(kPa)	Average water vapor pressure during the considered months
**WS	(m/s)	Average wind velocity in the environment where lizards occur
		
ALT_CV	%	Coefficient of variation of altitude
BIO4A		Temperature seasonality: Coefficient of variation in monthly temperature
BIO 15		Precipitation seasonality: Coefficient of variation in monthly precipitation
*RAD_CV		Coefficient of variation in monthly radiation
VAPR_CV		Coefficient of variation of water vapor pressure
WS_CV		Coefficient of variation of wind speed

A plus sign (+) indicates control variables, while an asterisk (*) denotes variables that were highly correlated and therefore excluded from the analyses.

^*^Excluded only for species with *Cytb* data.

^**^Excluded only for species with12*S rRNA* data.

^***^Excluded for species possessing data on both genetic markers.

*Explanatory variables* (Table 1):

#### Species traits

We compiled a suite of species-specific traits from the literature (e.g., Abdala et al. 2012; 2021a, 2021b among others; Supplementary S5) and our experience on the subject, including mean and maximum snout-vent length (SVL and SVL_max_), primary substrate use (arboreal, halophile, i.e., species that occur in salt terrain, psammophilous, saxicolous, terrestrial), diet (herbivorous, insectivorous, omnivorous), reproductive mode (oviparous, viviparous), and presence (or absence) of sexual dichromatism.

From IUCN portal (IUCN 2024), we registered threat status (DD, data deficient; EN, endangered; LC, least concern; NE, not evaluated; NT, near threatened; VU, vulnerable) as well as the presence of specific threats (agriculture expansion; energy production; natural; residential-commercial; viticulture settlements and transportation-service) and coded each as either “Yes” or “No.”

#### Geographic and abiotic data

We obtained geographic range data for 78 species from the IUCN portal (IUCN 2024). For species lacking distribution data in the IUCN data base (e.g., *Liolaemus antonietae*, *L. anquapuka*, *L. audituvelatus*, *L. campanae*, *L. crandallii*, *L. kulinko*, *L. meraxes*, *L. multiformis*, *L. nigrodorsum*, *L. splendidus*), we generated range shapefiles using QGIS v3.36.0 (QGIS.org 2024), incorporating species'occurrence records from herpetological collections (e.g., Colección de Herpetología IBIGEO, Instituto de Herpetología Fundación Miguel Lillo), original species descriptions, and relevant literature (Supplementary S3, S5; Abdala et al. 2021a, 2021b). We calculated species range area (km^2^) using QGIS and included it as a predictor variable (Table 1).

To characterize the environmental variability and heterogeneity across species' occurrence areas, while maximizing spatial representativeness, we used each species' full range distribution and generated 20 random geographic points within it. Using these points, we extracted 11 abiotic variables (Table 1) from Worldclim v2.1 database (Flick and Hijmans 2017) at a resolution of approximately 19.87 km^2^. All numeric variables (Table 1) were log_10_-transformed and standardized using the “scale” function, to achieve a mean of zero and unit variance after, we evaluated pairwise correlations (Pearson's *r* ≥ 0.60; *corrplot* package; Wei and Simko 2021; Supplementary S6). We then applied the “findCorrelation” function (cutoff = 0.6; *caret* package; Kuhn 2008) to identify and remove highly correlated predictors. The retained variables for species with *Cytb* data included: ALT (altitude, masl), BIO1 (annual mean temperature, °C), BIO7 (annual temperature range, °C), VAPR (water vapor pressure, kPa), and WS (wind speed, m/s) (see; Table 1). For species with *12S rRNA* data, the variables retained were similar, although RAD (radiation, W) and BIO12 (annual precipitation, mm^3^) were included, while VAPR and WS were excluded (Table 1).

We calculated the coefficient of variation (CV) for selected variables (ALT_CV, RAD_CV, VAPR_CV, and WS_CV). We also included BIO4A (temperature CV) and BIO15 (precipitation CV), excluding RAD_CV due to high correlation (Supplementary S6). The same procedure was applied for both genes.

To visualize geographic patterns in π values, we extracted centroid coordinates for each species based on their distribution range and mapped π values onto these points, distinguishing reproductive modes and taxonomic groups (Fig. 3).

**Figure 3:**
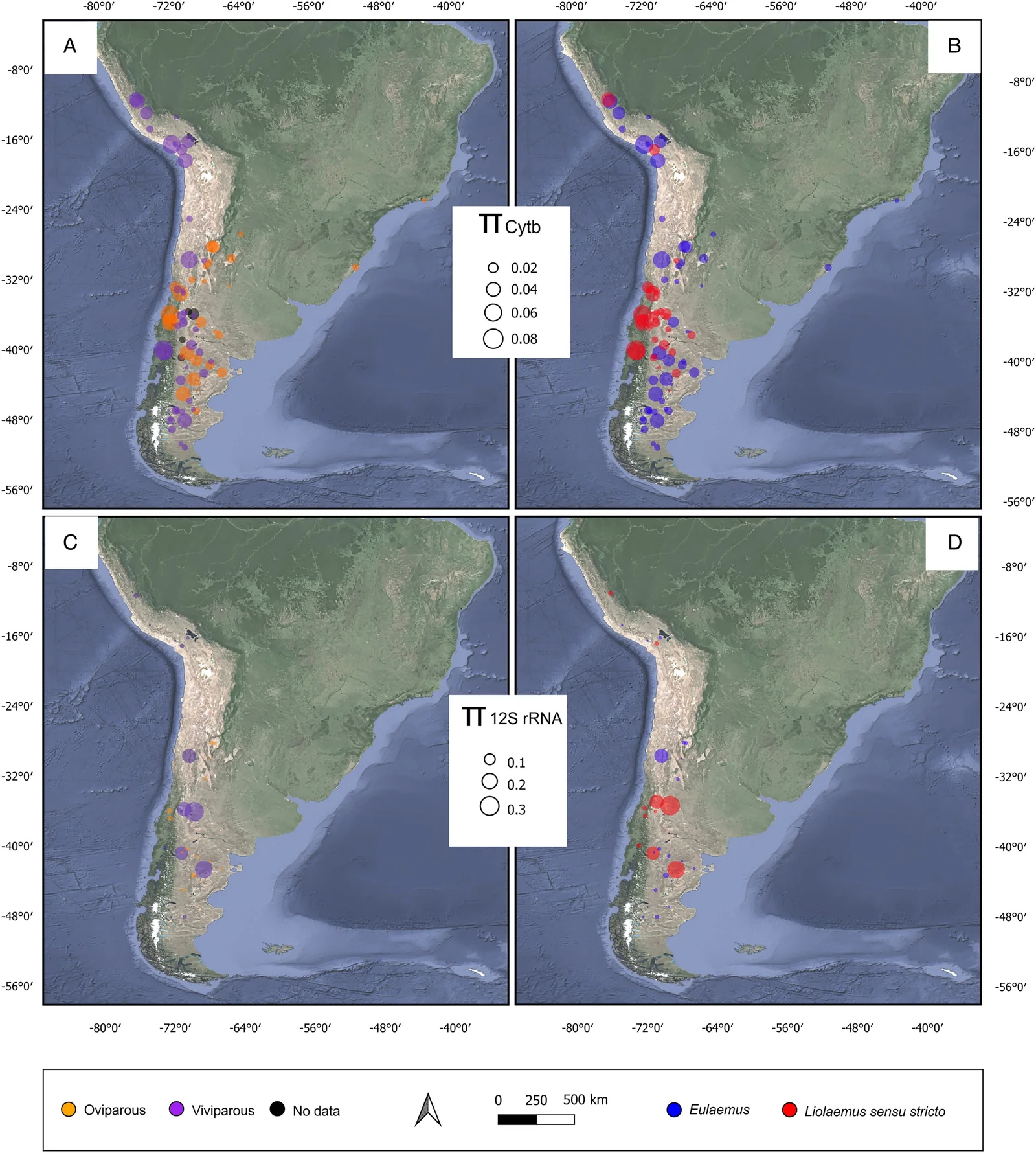
Geographic centroids of *Liolaemus* species displaying nucleotide diversity (π), values for *Cytb* (A-B) and *12S rRNA* (C-D). In each panel circle sizes within insert box represent π values. The left panels show species by reproductive mode (orange: oviparous, purple: viviparous), while the right panels show species by sub genus (red: *Liolaemus sensu stricto*, blue: *Eulaemus*). Map based on Google Satellite imagery. Data retrieved from [http://www.google.com/maps]. © Google. Figure edited using QGIS 3.36 software.

## Data analyses

### Isolation by geographic distance (IBD)

For each species, we calculated intraspecific geographic distances (km^2^) with “distm” (*geosphere* package; Hijmans 2022), based on the geographic coordinates of DNA voucher-donors extracted from associated literature (Supplementary S2). We compute also intraspecific genetic distances, which provide one measure of genetic differences among individuals, for *Cytb* and *12S rRNA* genes with “dist.dna” function (*ape* package; Paradis and Schliep 2019). This function computes a matrix of pairwise distances from DNA sequences using a model of DNA evolution (model = “K80”, Kimura 1980). After obtaining both matrices (geographic and genetic), we removed redundant values from each distance matrix using the “lower.tri” function; this gives only elements from the lower triangle of matrix. Mantel tests relating both data matrices were performed using “mantel.rtest” function (Pearson correlation, *ade4* package; Dray and Dufour 2007) with 9,999 permutations. Resulting correlation coefficients (*r*), *P*-values, and sample size (n) were recorded for each species. For species with non-significant *r* values (*P >* 0.05), *r* was set to zero. Additionally, we employed the MRM function (*ecodist* package; Goslee and Urban 2007) to assess slope of regression. MRM performs multiple regressions on distance matrices using permutation tests (nperm =10,000) to assess the regression coefficients. Geographic distance was fitted as log_10_ (Geographic distance +1), after this, we extracted species-specific IBD slopes (β_IBD_). Non-significant β_IBD_ (*P* > 0.05) were set to zero.

We then tested several explanatory variables; including species range area, ALT and SVL (log_10_-transformed and standardized with scale function), as well as substrate use, diet type, and reproductive mode (Table 1), as predictors of β_IBD_ values. Before analyses, we tested for phylogenetic signal of β using Blomberg´s *K*, (where values closer to zero indicate weak phylogenetic patterns; Blomberg et al. 2003) and determined the best-fitting model of evolution explaining β_IBD_ values (Brownian motion [BM], early-burst [EB], Ornstein-Uhlenbeck [OU]; Harmon et al. 2008, 2010) with “fitContinuous” (*geiger* package; Pennell et al. 2014; *ape* package Paradis and Schliep 2019). Model selection was made following two criteria: the delta Akaike's information criterion for small sample sizes (ΔAIC_C_) and Akaike's weight (AIC_C*Wi*_), whose better models are indicated by ΔAIC_C_ values less than 2 and the highest AIC_C*Wi*_ value (Burnham and Anderson 2002; Supplementary S7). β_IBD_ values (*Cytb* and *12S rRNA*) presented weak and non-significant phylogenetic patterns (Supplementary S7): β_IBD-*Cytb*_  *K* = 0.05, *P =* 0.06; β_IBD-*12S rRNA*_  *K* = 0.09, *P =* 0.38, and OU was the best evolutionary model for slopes of both genetic markers (Supplementary S7). Then, for analyses involving β_IBD_ values, we fitted an OU correlation structure using the “corMartins” function (*ape* package; Paradis and Schliep 2019) to account for phylogenetic patterns.

Afterward, we tested whether β_IBD-*Cytb*_ and β_IBD-*12S rRNA*_ were affected by bp and *n*. No significant effect of bp was detected (*P* = 0.16 and *P* = 0.4, respectively), whereas *n* significantly influenced both markers (*P* = 0.02 and *P* < 0.01, respectively). Given that sample size affected β_IBD_ values across both genetic markers, we accounted for *n*, phylogenetic structure, and zero inflation (the latter inferred from non-significant β_IBD_ estimates; Supplementary S8). We employed a generalized additive models for location, scale, and shape (GAMLSS) framework, which enables the fitting of multiple regression models to variables following a zero-inflated gamma distribution, while accounting for multiple covariates. For β_IBD-*Cytb*_, we fitted 63 models combining six predictor variables (see above; Supplementary S8), whereas for β_IBD-*12S rRNA*_, 31 models were fitted using five variables (Supplementary S8). The substrate use factor was excluded from the latter due to asymmetries in species representation across its levels (Supplementary S8), which prevented meaningful comparisons. All models were fitted using the *gamlss* package (Rigby and Stasinopoulos 2005), specifying control parameters (sigma.formula = ∼ n; nu.formula = ∼ predictor variables; family = ZAGA; correlation = OU correlation). Model selection was based on ΔAICc and AICc*_Wi_* criteria. These GAMLSS models were subsequently evaluated using 1,000 bootstrap permutations, with 95% confidence intervals estimated from the 2.5th and 97.5th percentiles, corresponding to the “lower_2.5” and “upper_97.5” bounds.

To assess IBD and its relationship with body size, we standardised and transformed *r* values from Mantel test into their effect sizes (*Zr r* and Var*Zr r*, accounting for variation in *n*) using the “escalc” function (*metafor* package; Viechtbauer 2010). We then applied a mixed-effects regression model using the “rma.mv” function from *metafor* (Viechtbauer 2010) to examine the relationship between *Zr r* and SVL. SVL was log_10_-transformed, standardized using the scale function, and included as a fixed effect. Phylogeny was incorporated as a random effect through a correlation matrix (A) derived from the phylogenetic tree (Fig. 2) using the “vcv” function (*ape* package; Paradis and Schliep 2019).

### Genetic diversity

We examined whether π values for *Cytb* and *12S rRNA* were explained by body size, life-history traits, climatic variables, and environmental heterogeneity (Table 1). For this, we fitted Bayesian generalized linear mixed models (GLMMs) using “brms” (Bürkner 2017), specifying a Gamma distribution with a log link. Phylogenetic covariance among species was incorporated via a random effect structure (details of the models' structure in Supplementary S8). The existence of an effect was determined based on the maximum probability of effects (MPE ≥ 0.90), which represents the posterior probability that a given coefficient is strictly positive or strictly negative (Makowski et al. 2019). We explored the effect of each individual predictor on π, while also including control variables and phylogenetic structure in the models (Supplementary S8). Model comparison was conducted within a Bayesian framework using the expected log pointwise predictive density (*elpd*), estimated via leave-one-out cross-validation (Vehtari et al. 2017) and implemented in the *loo* package (Vehtari et al. 2025). Models were ranked by *elpd* scores (higher values indicate better predictive performance), and a model was considered less informative than the best one if its relative difference (Δ*elpd*), plus the associated standard error (SEΔ*elpd*), did not include zero. Models were run with the following settings: chains = 4, cores = 4, iter = 110,000, warmup = 3,000, thin = 100, and adapt_delta = 0.95. Convergence was assessed. Posterior draws were summarized using the maximum a posteriori (MAP) estimate and the 50% and 95% highest density intervals (HDI_50_; HDI_95_) (Makowski et al. 2019).

Prior, we tested the dependence of π values on bp and *n*; results indicated that, for π-*Cytb*, *n* (MAP = 0.23, MPE = 0.97) had a considerable influence, whereas bp (MAP =0.07, MPE = 0.750) did not, given its low MPE (< 0.9) and weak MAP. In contrast, for π-*12 s rRNA*, bp did show a substantial effect (MAP = −0.91, MPE = 0.99), whereas *n* did not (MAP = −0.04, MPE = 0.57). Therefore, we controlled for *n* in π-*Cytb* and for bp in π-*12S rRNA* analyses.

Due to missing data for some variables, we performed two sets of analyses (details can be observed in Supplementary S8): (1) one based on seven numeric variables and a larger number of species (88 for *Cytb* and 37 *12S rRNA*), testing seven models each (one per variable) and (ii) another set incorporating five categorical variables (e.g., reproductive mode, substrate use) with fewer species (58 for *Cytb* and 29 for *12S rRNA*), testing 12 models each. Additionally, we tested the five coefficients of variation in five models. All candidate models and full parameter estimates are presented in Supplementary S8. In the main text, we report only predictors with strong posterior support (MPE ≥ 0.90), as these represent the most robust associations with genetic diversity metrics. Finally, we examined whether π values were associated with β_IBD_ using similar GLMMs model.

## Results

### DNA sequence retrieval

A GenBank search for “*Liolaemus*” yielded 14,201 entries, of which 14,133 contained *Liolaemus* sequence data corresponding to 238 described species and 96 putative/undescribed species (cf. or sp.; Supplementary S1). The most frequently studied genetic marker was *Cytochrome b* (*Cytb*; 5,387 sequences), followed by the *12S ribosomal RNA* (*12S* rRNA; 1,175 sequences). Other frequently represented markers included *Kinesin family member 24* (*KIF24*; 689 sequences), *Exophilin 5* (*EXPH5*; 525 sequences), *Oocyte maturation factor Mos* (*C-mos*; 467 sequences), and *Prolactin receptor* (*PRLR*; 461 sequences; Supplementary S1). Given the high representation of *Cytb* and *12S rRNA* sequences, we focused our analyses on these two markers. Initially, *Cytb* data were available for 203 described and 60 undescribed *Liolaemus* species. After filtering, 3,295 sequences representing 88 described species were retained (Figs. 1 and 2A). For *12S rRNA*, data were available for 170 described and 61 undescribed species, of which 500 sequences from 37 described *Liolaemus* species remained after filtering (Figs. 1 and 2B). In both cases, species with insufficient or geographically unresolvable data were excluded from specific analyses.

### Isolation by distance (IBD) patterns

#### Frequency of IBD across species

For *Cytb*, IBD was tested in 74 species (Fig. 2A), because 14 species had samples from a single geographic location; therefore, we could not calculate their geographic distance matrices. Among these, 41 species (55.40%) exhibited significant Mantel correlations (*r*) that ranged from 0.105 (*Liolaemus koslowskyi*) to 0.990 (*L. pseudoanomalus*), with a mean of 0.554. For *12S rRNA*, IBD was tested in 33 species. Of these, 14 species (42.42%) showed significant Mantel *r* values, ranging from *r* = 0.205 in *L. buergeri* to 0.955 in *L. pseudoanomalus* (Fig. 2B).

#### IBD and body size (SVL): mantel's r analysis

For *Cytb*, an analysis including 74 species (with significant *r* values retained and non-significant values set to zero) revealed no significant association between IBD (*r*) and SVL (Table 2). However, when considering only cases with significant *r* values (41 species), a significant negative association was observed (Table 2; Fig. 4), indicating that larger species tend to exhibit weaker IBD effects. For *12S rRNA*, no significant association between *r* and SVL was found, either considering all 33 species or only those with significant IBD *r* values (Table 2).

**Figure 4:**
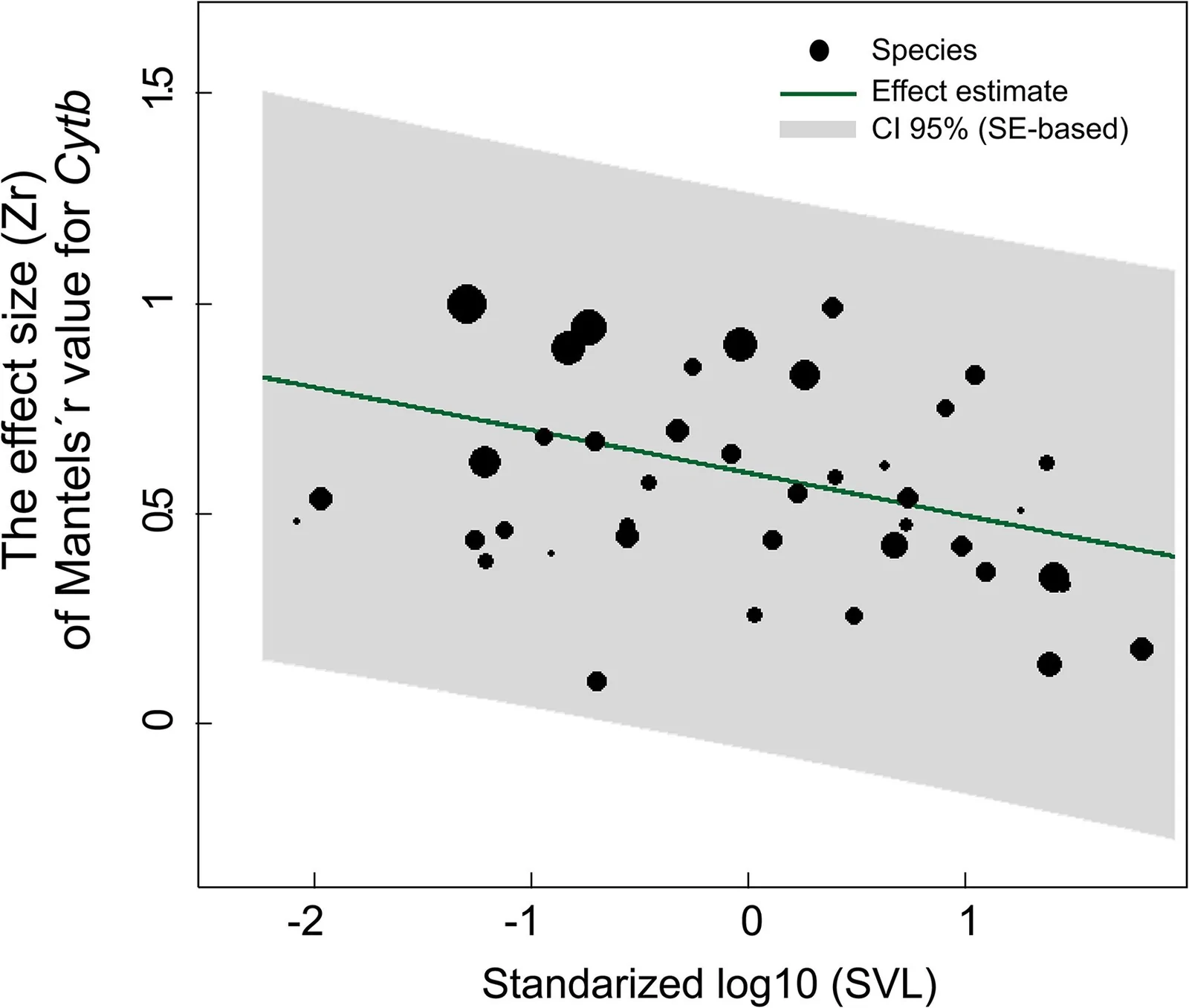
Negative association between the effect size of Mantel's correlation coefficient (*r*) for isolation by distance (IBD) and snout-vent length (SVL). This analysis includes only species with significant IBD (*Zr r* > 0). The size of the points represents both the variability associated with the effect size (*Zr*) and the relative weight of each species in the analysis. Larger points correspond to data with lower variability and greater influence on the regression results.

**Table 2:** Model parameters for the association between isolation-by-distance slope (β_IBD_) and species traits/environment for mitochondrial *Cytb* and *12S rRNA* datasets.

Gen	Model description	N spp.	Estimator (df1, df2)	Intercept	Predictor	Slopepredictor	*P_predictor_*	*P _Model_*
	IBDComplete _(*r* zero_ _+_ *_r_* _significants)_∼SVL	74	*F* = 3.30 (1,72)	0.54	SVL	−0.08	0.07	0.07
*Cytb*	*IBDPartial *_r_* _significants_ ∼SVL	41	*F* = 9.08 (1,39)	0.59	SVL	−0.10	<0.01	<0.01
	*β_IBD-*Cytb*_ ∼ AREA + ALT + SVL + SU + Diet type + RM	59	*LRT* = 60.92 (4,20)	−4.87	AREA	0.15	0.18	<0.001
					ALT	0.82	<0.01	
					SVL	−0.50	<0.01	
	*β_IBD_-*_Cytb_* ∼ ALT + SVL + SU + Diet type + RM	59	*LRT* = 56.12 (4,18)		ALT	0.76	<0.01	<0.001
					SVL	−0.50	<0.01	
	IBDComplete _(*r* zero_ _+_ *_r_* _significants)_∼SVL	33	*F* = 0.83 (1,31)	0.47	SVL	0.06	0.36	0.36
	IBDPartial *_r_* _significants_∼SVL	18	*F* = 1.46 (1,16)	0.65	SVL	0.07	0.24	0.24
	*β_IBD-*12S rRNA*_ ∼ ALT + SVL + Diet type	27	*LRT* = 21.20 (4,10)	−5.45	**ALT**	0.61	0.03	<0.01
		
*12S rRNA*					SVL	−0.04	0.90	
	*β_IBD-*12S rRNA*_ ∼ ALT		*LRT* = 13.11 (4,6)	−5.58	**ALT**	0.65	0.02	<0.01
	*β_IBD-*12S rRNA*_ ∼ ALT + SVL + Diet type + RM		*LRT* = 24.83 (4,12)	−4.57	**ALT**	0.49	0.04	<0.01
					SVL	0.80	0.16	
	*β_IBD-*12S rRNA*_ ∼ ALT + SVL		*LRT* = 16.27 (4,8)	−5.66	**ALT**	0.64	0.02	<0.01
					SVL	−0.20	0.35	
	*β_IBD-*12S rRNA*_ ∼ AREA + ALT		LRT = 18.82 (4,8)	−5.67	AREA	0.20	0.65	<0.01
					**ALT**	0.68	0.02	
	*β_IBD-*12S rRNA*_ ∼ SVL + Diet type + RM		LRT = 19.51 (4,10)	−4.65	SVL	1.10	0.07	<0.01

The table presents model description; number of species analyzed (Nspp.); Estimator (*F*-statistic or likelihood ratio test) comparing the full ZAGA model to a null model (LRT) with its degrees of freedom (df); intercept; predictor; slope; predictor *P* -values, and *P*-values of models. Asterisk (*) indicates those statistically significant models (*P* < 0.05). Mean differences among factor levels (substrate used, diet type, and reproductive mode) are not shown.

#### Determinants of the IBD slope

For *Cytb*, β_IBD-*Cytb*_ was calculated for 74 species. Among these, 53 species (71.62%) exhibited significant β_IBD-*Cytb*_ (Supplementary S9). Values ranged from 0 to 0.017 (with the maximum observed in *Liolaemus monticola*). Two best-fitting models were identified for β_IBD-*Cytb*_ estimates (Table 2; Supplementary S8). Both models showed a significant positive association between β_IBD-*Cytb*_ and altitude, and a negative association with SVL (Table 2; Figs. 5A–B). The effect was weaker in insectivorous species than in omnivorous species (*t* = 2.16, *P* < 0.05), and in viviparous species compared to oviparous ones (*t* = −2.78, *P* < 0.001). In the second-best model, substrate use was significant, with arboreal species exhibited stronger β_IBD*-Cytb*_ slopes than terrestrial species (*t* = −2.13, *P* < 0.05). After bootstrap permutations, only altitude and SVL remained significant in both models (*P* < 0.05). For altitude, the mean slope was 0.75 (*bootstrap permutations* 95% CI [0.32, 1.17]). For SVL, the mean slope was −0.52 (*bootstrap permutations* 95% CI [−1.11, −0.11]). All other predictors were not statistically significant (*bootstrap permutations P* > 0.05).

**Figure 5:**
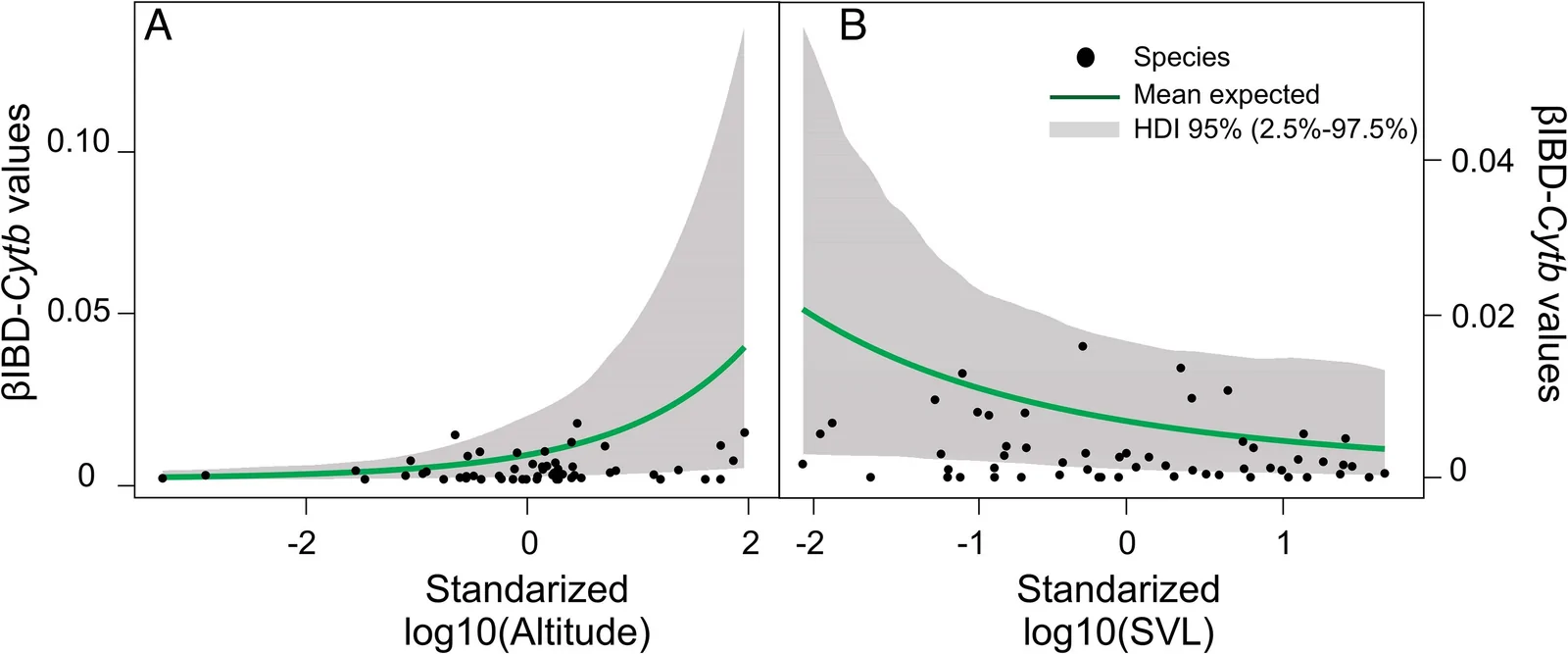
(A) Positive and (B) negative associations between the *Cytb* IBD slope (β) and Altitude and Snout–Vent Length (SVL), respectively. The green line shows the model-predicted values, while the black dots represent the observed data by species. We show (grey) the empirical distribution of observed values alongside model-predicted means and their 95% bootstrap confidence intervals.

For *12S rRNA*, β_IBD-*12S rRNA*_ values were significant in 16 out of the 34 tested species (47.05%; see Supplementary S9). Values ranged from 0 (17 species) to 0.13 in *Liolaemus austromendocinus*, which was considered an outlier and excluded from subsequent analyses. After excluding the outlier, the maximum β_IBD-*12S rRNA*_ was 0.0065 in *L. boulengeri* and six best-fitting models were identified (Table 2; Supplementary S8), in which altitude consistently emerged as a relevant predictor. Altitude showed a positive association with β_IBD-*12S rRNA*_ (Table 2). However, the sixth model (β_IBD-*12SrRNA*_∼SVL + RM + Diet type) did not include altitude, and instead revealed a significant difference between omnivorous and insectivorous species (*t* = −2.27, *P* < 0.05), with the β_IBD-*12S rRNA*_ effect smaller in omnivorous than in insectivorous species. However, after bootstrap permutations none of these predictors were statistically significant (*bootstrap permutations P* > 0.05).

### Genetic diversity (π) estimates

Across the 88 species π-*Cytb* ranged from 0 (*Liolaemus hermannunezi*) to 0.225 (*L. cuyanus*), mean = 0.021. Since *L. cuyanus* contributed disproportionately large to the variance (57.64%), and *L*. *hermannunezi* has a π value of zero, they were excluded from further analyses. Without these species, π-*Cytb* values in the remaining 86 ranged from 0.00078 (*L. burmesteri*) to 0.081 (*L. cyanogaster*), with a mean of 0.019. Geographically, species were more broadly distributed latitudinally (from 11°S *Liolaemus walkeri* to ∼ 51°S *L. sarmientoi*; Figs. 3A–B) than longitudinally (42°W *L. lutzae* to ∼ 75°W *L. walkeri*). Longitudinal geographic extension showed a credible negative probabilistic effect on π-*Cytb* (MPE = 0.98, MAP = −0.07 [−0.12, −0.01]). A differentiation was observed between the two clades (MPE = 0.99): species belonging to the *Liolaemus sensu stricto* subgenus exhibited an increase in π-*Cytb* values from East to West (MPE = 0.99, MAP = −0.28 [−0.48, −0.09]). In contrast, latitudinal extension revealed a less credible probability of effect (MPE = 0.91, MAP = 0.02 [−0.01, 0.05]), with values increasing toward the tropics. No clear differentiation was observed between reproductive modes or subgenus, as all associated effects showed lower than 0.90 values of MPE.

For *12S rRNA*, π values ranged from 0 (*Liolaemus darwinii*, *L. hermannunezi*, *L. lentus*) to 0.32 (*L. austromendocinus*). These three species with zero π values were excluded from further analyses. After their removal, π values for *12S rRNA* among the remaining 34 species ranged from 0.0006 (*L. antumalguen*) to 0.318 (*L. austromendocinus*), with a mean of 0.0396. Species were broadly distributed from 11°S (*L. walkeri*) to 48°S (*L. hatcheri*; Figs. 3C–D) and from 65°W (*L. melanops*) to 75°W (*L. walkeri*; Figs.3C–D). The highest π-*12S rRNA* values were found in southern Argentina, particularly in four *Liolaemus sensu stricto* species (*L. austromendocinus*, *L. buergeri*, *L. kriegi*, *L. petrophilus*; Fig. 3D). π-*_12S rRNA_* did not show MPE values greater than 0.90 when associated with latitude and longitude.

### Determinants of genetic diversity (π)

#### All predictors, numeric and categorical variables

For π-*Cytb*, when considering all explanatory variables (*n* = 58 species), the twelve fitted models could be considered informative based on the Δ*elpd* criterion (Supplementary S8). Based on Δ*elpd* and the highest probability of direction (pd) values (pd ≥ 0.90), three models (and variables) showed the highest posterior consistency: annual temperature range (BIO7), SVL, and species range area (AREA). Among these, SVL presented a MPE value equal to 0.90 (Bayes R² = 0.45) with a negative effect on π-*Cytb* (MAP = −0.23 [−0.51, 0.12]; Table 3). Whereas BIO7 and AREA showed MPE values lower than 0.90 (MPE_BIO7_ = 0.89; MPE_AREA_ = 0.84). No MPE values exceeded 0.90 when considering reproductive mode or subgenus.

**Table 3:** Summary of the main results from Bayesian GLMMs, including only predictors with strong posterior support (MPE ≥ 0.90) for π in both genes: Cytb and 12S rRNA. Model details are provided in the Supplementary S8.

Predictor	Group analyzed	π	N spp.	MPE	MAP	Support
Latitudinal extension	All species		86	0.91	0.02 [−0.01, 0.05]	✓
Longitudinal extension	All species		86	0.98	−0.07 [−0.12, −0.01]	✓✓
Longitudinal extension	*L. ss* *stricto*		35	0.99	−0.28 [−0.48, −0.09]	✓✓
AREA	All species		86	0.91	0.16 [−0.07, 0.38]	✓
BIO7	All species (All predictors)		58	0.9	− 0.23 [−0.51, 0.12]	✓
BIO7	All species (Numeric predictors)		86	0.97	−0.27 [−0.53, 0.01]	✓✓
BIO7	Vivip. species		43	0.99	−0.66 [−1.00, −0.20]	✓✓
BIO7	*L. ss stricto*		35	0.98	−0.50 [−0.91, −0.05]	✓✓
SVL	All species	*Cytb*	86	0.93	− 0.19 [−0.42, 0.05]	✓
BIO4A	All species		86	0.97	−0.25 [−0.53, 0.01]	✓✓
β_IBD-*Cytb*_	All species		73	0.99	0.29 [0.07,0.48]	✓✓
BIO1	All species (All predictors)		27	0.99	−0.58 [−1.05, −0.12]	✓✓
BIO1	All species (Numeric predictors)		3427	0.92	−0.3 [−0.86, 0.13]	✓
BIO1	Eul.	12S rRNA	20	0.98	−0.52 [−1.12, −0.03]	✓✓
βIBD-12S rRNA	All species		29	0.99	0.46 [0.14,0.77])	✓✓

The table includes predictor variables, group analyzed (All species = analysis including all species without subdivision; *L. ss stricto* = species belonging to the *Liolaemus sensu stricto subgenus*; *Eul*. = species belonging to the *Eulaemus* subgenus; Vivip. = species with viviparous reproductive mode), sample size (*N* = number of species analyzed), MAP (maximum a posteriori estimate), and the 50% and 95% highest density intervals (HDI50; HDI95). Symbols indicate posterior support: ✓ = MPE ≥ 0.90; ✓✓ = MPE ≥ 0.95.

For π-*12S rRNA*, 12 models including both numeric and categorical variables (*n* = 27 species) were evaluated. Only one model showed the best fit (Δ*elpd* = 0; Bayes R² = 0.76): π-*12S rRNA* ∼ annual mean temperature (BIO1, MPE = 0.99; Supplementary S8, Table 3), which showed a negative association with π-*12S rRNA* (MAP = −0.58 [−1.05, −0.12]; Table 3). No MPE values exceeded 0.90 when considering reproductive mode or subgenus.

#### Numeric predictors

For π-*Cytb*, considering only numeric variables (86 species), seven fitted models were considered informative based on the Δ*elpd* criterion (Supplementary S8). Based on pd values, 3 models showed the highest posterior consistency: BIO7, SVL, and AREA (MPE_BIO7_ = 0.97, Bayes *R*² = 0.37; MPE_SVL_ = 0.93, Bayes *R*² = 0.37; MPE_AREA_ = 0.91, Bayes *R*² = 0.37; Table 3). BIO7 and SVL exhibited negative effects (MAP_BIO7_ =−0.27 [−0.53, 0.01], Fig. 6A; MAP_SVL_ = −0.19 [−0.42, 0.05], Fig. 6D), while AREA showed a positive effect (MAP_AREA_ = 0.16 [−0.07, 0.38], Fig. 6E) on π-*Cytb*. For BIO7, when comparing reproductive modes, there were credible differences (MPE = 0.96): viviparous species showed a credible and negative association with BIO7 (MAP = −0.66 [−1.00, −0.20], MPE = 0.99, Fig. 6B; Supplementary S8). Between the two subgenera, credible differences were detected (MPE = 0.91). Only species belonging to the *Liolaemus sensu stricto* subgenus exhibited a decrease in π-*Cytb* values with increasing BIO7 (MAP = −0.50 [−0.91, −0.05], MPE = 0.98, Fig. 6C; Supplementary S8). Considering environmental heterogeneity (derived from different climatic coefficients of variation), the model including temperature seasonality (BIO4A) provided the best fit (Δ*elpd* = 0, SE = 0, Bayes *R*² = 0.39), although the other models remain viable alternatives (Supplementary S8). Only BIO4A exhibited both a high posterior probability (pd = 0.96) and a high maximum probability of effect (MPE = 0.97), along with a negative effect on π-*Cytb* (MAP_BIO4A_ = −0.25 [−0.53, 0.01]; Fig. 6F). No MPE values exceeded 0.90 were observed between the two subgenera or reproductive modes.

**Figure 6:**
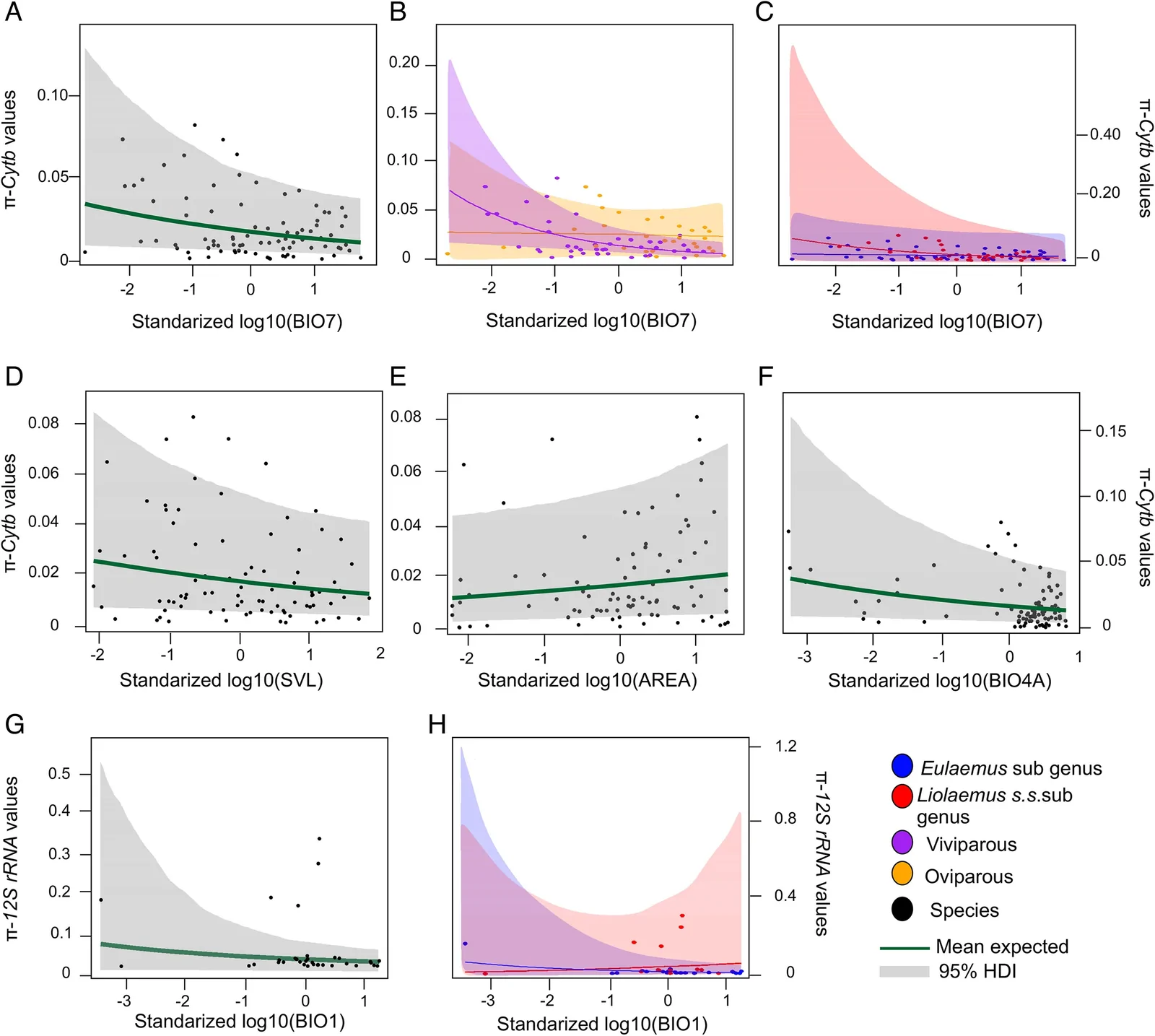
Results from Bayesian GLMMs Models showing predictors with a Maximum Probability of Effects (MPE ≥ 0.90) on nucleotide diversity (π). Slopes represent posterior mean predictions, dots indicate observed data, and the shaded areas denote 95% Highest Density Intervals (HDIs). Panels (A) to (C) illustrate the effect of annual temperature range (BIO7) on π-*Cytb*. Panel (D) shows the effect of Snout–Vent Length (SVL), while panels (E) and (F) display the effects of species range area (AREA) and temperature seasonality (BIO4A) on π-*Cytb*, respectively. Panels (G) and (H) present the effect of annual mean temperature (BIO1) on π-*12S rRNA*. Blue and red points represent species belonging to the *Eulaemus* and *Liolaemus*  *sensu stricto* subgenera, respectively. Orange and purple points indicate oviparous and viviparous species, respectively.

For π-*12S rRNA* (34 species), four models could be considered best-fitted (Supplementary S8), however, only the model with BIO1 (Bayes R² = 0.37) showed an MPE value greater than 0.90 (0.92), with a negative effect on π-*12S rRNA* (MAP_BIO1_ = −0.3 [−0.86, 0.13]; Fig. 6G). A difference was detected between the two subgenera (MPE = 0.92): only species from the *Eulaemus* subgenus showed a negative and credible effect (MAP = −0.52 [−1.12, −0.03], MPE = 0.98; Fig. 6H). No credible differences were observed between the two reproductive modes (MPE = 0.76). Regarding environmental heterogeneity, four models could be considered. However, none of these models exhibited an MPE greater than 0.9 (Supplementary S8).

#### Relationship between β_IBD_ and π

π-*Cytb* showed a credible (MPE = 0.99) and positive association (MAP = 0.29 [0.07, 0.48]) with β_IBD-*Cytb*_ values (Fig. 7A), suggesting that species exhibiting stronger IBD patterns also tend to have higher nucleotide diversity on *Cytb* gene.

**Figure 7:**
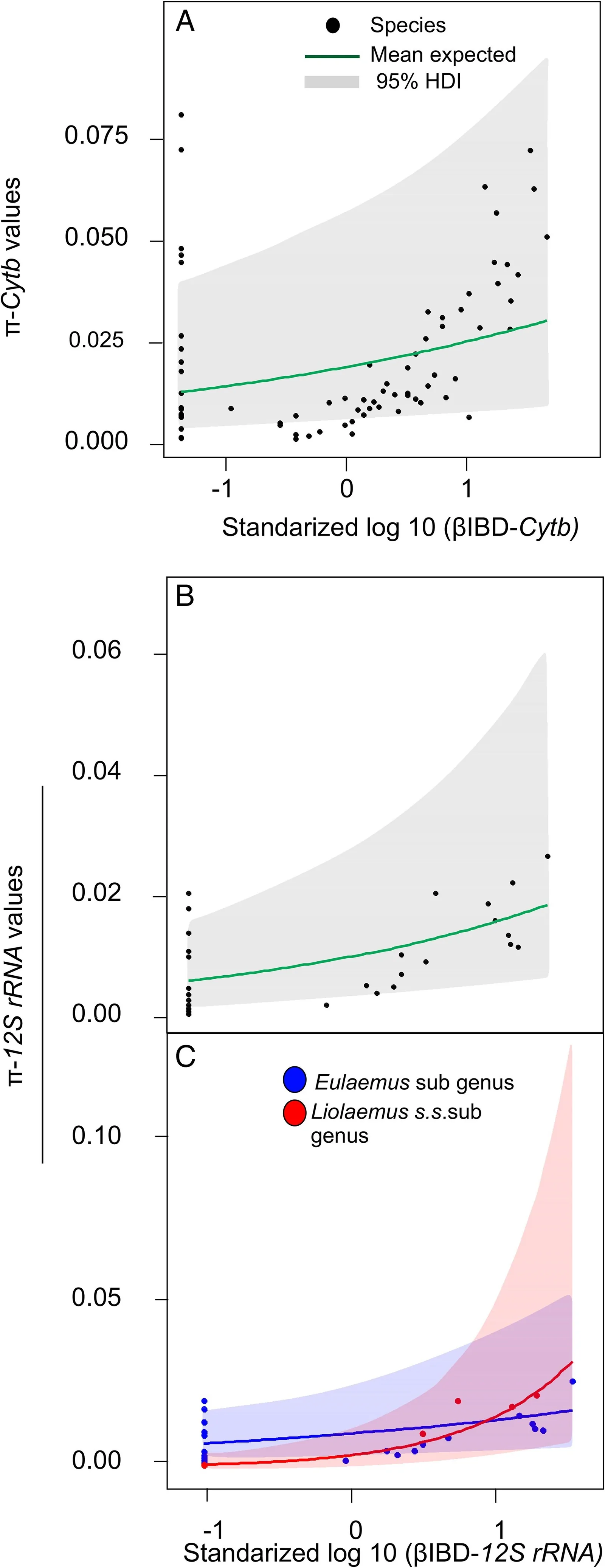
Results from GLMMs models showing associations with a Maximum Probability of Effects (MPE ≥ 0.90) between the Isolation by Distance (IBD) slope (β) and nucleotide diversity (π). Slopes represent posterior mean predictions, dots indicate observed data, and shaded areas denote 95% Highest Density Intervals (HDIs). Panel (A) shows the effect of β_IBD-*Cytb*_ on π-*Cytb*, while panels (B) and (C) display the effect of β_IBD-*12S rRNA*_ on π-*12S rRNA*. The latter figure highlights differences between subgenera. Blue and red points represent species belonging to the *Eulaemus* and *Liolaemus* sensu stricto subgenera, respectively.

No credible MPE values were observed testing association π-*12S rRNA* and β_IBD-*12S rRNA*_, however, after removing four outliers, there was a credible (MPE = 0.99) and positive (MAP = 0.46 [0.14, 0.77]; Fig. 7B) association. Similar results were observed only in species belonging to *Liolaemus sensu stricto* subgenus (MPE = 0.99; MAP = 1.41[0.69, 2.08]; Fig. 7C). No credible MPE values were observed between reproductive modes in both genetic markers and taxonomic groups (in case of π-*Cytb*).

## Discussion

Our study offers new clues about mitochondrial genetic diversity (π) in *Liolaemus* lizards, including its environmental (climate variability and habitat heterogeneity) and phylogenetic correlates. IBD was commonly observed and showed a credible positive association with π in *Cytb* and *12S rRNA* markers.

Although its prevalence across the genus and its association with specific predictors varied between genes, altitude and SVL consistently predicted IBD for *Cytb*.

In the case of *12S rRNA*, similar predictors were identified by the models as potentially relevant, but none were supported as significant, as bootstrap-based prediction intervals revealed substantial uncertainty, indicating that these effects may not be robust across resampled datasets. Similar patterns were observed for categorical variables (diet type, reproductive mode, and substrate use) in both genes. The π in the two mitochondrial markers was negatively influenced by different factors depending on the gene studied. For *π-Cytb*, thermal variation (annual temperature range) and thermal heterogeneity (temperature coefficient of variation) were relevant, whereas mean annual temperature (BIO1) seemed more relevant for explaining π-*12S rRNA*. Comparable patterns held within reproductive modes (oviparous vs. viviparous) and across subgenera (*Eulaemus* vs. *Liolaemus sensu stricto)*, suggesting a potential interplay between evolutionary history and climatic factors in shaping the genetic diversity of *Cytb* and *12S rRNA* genes in *Liolaemus* lizards.

### Determinants of isolation by distance

IBD was relatively common across *Liolaemus*, with 55.4% of *Cytb* and 42.4% of *12S rRNA*-tested species exhibiting significant IBD. Altitude consistently emerged as a relevant predictor supporting IBD in *Cytb* and showed a suggestive trend in *12S rRNA*. These findings are broadly consistent with previous studies suggesting that geographical isolation, topographic barriers, and allopatric fragmentation, often linked to Andean uplift, have played a substantial role in promoting genetic diversity within Liolaemidae (Esquerré et al. 2019). Nonetheless, IBD patterns in *Liolaemus* lizards appear to be variable, ranging from clear to absent or mixed IBD patterns (e.g., Morando et al. 2007; Vera-Escalona et al. 2012; Villamil et al. 2017; Cab-Sulub and Álvarez-Castañeda 2022; Sánchez et al. 2024), similar to patterns reported across the Squamate order (Jenkins et al. 2010). Interestingly, larger body size was associated with weaker β_IBD_. Larger species tend to exhibit greater home ranges and dispersal capabilities (Perry and Garland Jr, 2002), which may attenuate the impact of geographic isolation on gene flow.

GAMLSS models exploring for β_IBD_ predictors indicated different trends in both genes that could not be recovered after applying the bootstrapping approach. This suggests that life-history and ecological traits could be important variables to explain β_IBD_ or perhaps they were overfitted by models due to asymmetry in the sample size within levels of factors. For example, we observed differences between arboreal and terrestrial species, with a potentially stronger β effect in the former. However, only four analyzed species utilized arboreal substrates compared to 27 terrestrials. Interpretations must be considered with caution due to the limited sample size, especially for *12S rRNA*, where only 16 species showed significant β values in our dataset. Also, the need to control for voucher sample size per species when analyzing β_IBD-*12S rRNA*_, highlighting a potential dependence on sampling effort. A meta-analytical study across multiple taxa (Jenkins et al. 2010) similarly reported that β estimates from IBD analyses are generally sensitive to sample size. Furthermore, differences in sampling localities should be acknowledged: while GenBank sequences often originate from multiple locations, certain species exhibited geographically restricted sampling (Supplementary S2). This constitutes a limitation that may influence the observed patterns.

Regardless of the type of genetic marker, we also observed a positive association between the strength of IBD (β_IBD_) and genetic diversity (π), suggesting that stronger spatial structure may be associated with higher within-species diversity, particularly for *Cytb*. Geographic distance has been shown to influence genetic differentiation in a variety of organisms, including lizards (Fonseca et al. 2024; McGreevy Jr et al. 2024) as well as other taxa (Li et al. 2021; Baccaro et al. 2024). This broadly observed pattern suggests that geographic distance may play a key role in shaping genetic diversity across species.

### Environmental determinants of genetic diversity

For both markers, the Ornstein–Uhlenbeck (OU) model provided the best fit, consistent with patterns reported for squamates (Larkin et al. 2023). The *12S rRNA* locus also showed support for a Brownian motion model, suggesting that neutral processes may partly shape its variation (Blomberg et al. 2003). This suggests that selective pressures may shape the nucleotide diversity of these two mitochondrial genes toward one or more evolutionary optima (Martins and Hansell 1997; Harmon et al. 2008, 2010). However, it is important to note that factors such as limited taxa sampling or data inconsistencies can lead to overestimation of OU processes in certain cases (Cooper et al. 2016).

Thermal variation (annual temperature range; BIO7) and heterogeneity (temperature coefficient of variation; BIO4A) influenced π-*Cytb*, while mean annual temperature (BIO1) influenced π-*12S rRNA*. The effect of thermal variation was stronger in viviparous species, inhabiting colder, harsher Andean and austral environments, where efficient thermoregulation and higher respiratory rates may have influenced *Cytb* evolution (Clavijo-Baquet et al. 2022; Cruz et al. 2022; Penman et al. 2022). An increase in mean annual temperature (BIO1) appeared to negatively influence π-*12S rRNA* more markedly in species belonging to the *Eulaemus* subgenus, which are broadly distributed across both latitudinal (North–South) and longitudinal (East–West) gradients (Abdala et al. 2021a). In this context, such broad spatial coverage may expose *Eulaemus* species to diverse thermal environments, and warmer climates could act as selective filters on mitochondrial diversity. This may contribute to the lower π-*12S rRNA* values observed, although the underlying mechanisms remain to be clarified.

Similar environmental determinants of genetic diversity have been reported in amphibians and in other taxa (Amador et al. 2024; Sexton et al. 2014), reinforcing those climatic and environmental gradients are key drivers of intraspecific genetic variation in ectotherms. We also observed that longitudinal range had a credible negative effect on π-*Cytb*. *Liolaemus* species exhibit a western distribution along Pacific coast of Peru and Chile, extending eastwards to reach the Atlantic coast of Uruguay and southeastern Brazil (Abdala et al. 2021a, 2021b). Our results may have been influenced by two eastern outliers, *Liolaemus lutzae* and *L. occipitalis* (Fig. 3), which display an extremely eastern distribution (Brazilian coast) and low π-*Cytb* values (0.004 and 0.011, respectively). Nonetheless, a similar trend was apparently observed within the *Liolaemus sensu stricto* subgenus, which tend to exhibit higher π values among western-distributed taxa. Between 72° and 69° W, relatively high *π-Cytb* values were observed (mean π = 0.046), from *L. cyanogaster* (π = 0.08101 at 72.63° W) to *L. gununakuna* (π = 0.02361 at 69.40° W). Many species within this range (*L. chiliensis*, *L. cyanogaster*, *L. gununakuna*, *L. lemniscatus*, *L. meraxes*, *L. monticola*, *L. nitidus*, *L. pictus*, *L. tacnae*, *L. tenuis*, *L. walkeri*) have complex phylogeographic histories, isolated populations and natural barriers that likely promote genetic structuring (Victoriano 2020). These species also have complex taxonomic histories, including cases of synonymization, re-descriptions, and documented hybridization processes (e.g., Medina et al. 2015, 2017; Olave et al. 2018; Araya-Donoso et al. 2019; Morando et al. 2020; Quinteros et al. 2020; Abdala et al. 2021a, 2021b). Although taxonomic revisions reflect historical uncertainty rather than biological processes per se, hybridization and the presence of cryptic or recently diverged lineages could contribute to the elevated π-*Cytb* values observed in these western taxa. Further investigation is required to disentangle the relative contributions of these factors.

In summary, our results suggest the presence of shared patterns across both mitochondrial markers. IBD is relatively common in this genus and it is associated with higher nucleotide diversity (π) in both mitochondrial genes. Thermal environmental gradients could also act as selective filters on intraspecific diversity. In the case of *Cytb* specifically, altitude appears to favor IBD occurrence and perhaps, the effects of IBD may be attenuated in larger species, possibly by modulating dispersal capacity. However, our characterization of within-species variation is based solely on two mitochondrial markers. Future research should conduct comprehensive analyses of genetic diversity across multiple genomic regions within each species to improve our understanding of evolutionary processes in *Liolaemus* lizards. Such studies should also incorporate population-genetic parameters accounting for mutation rate, genetic drift, directional selection, and linked selection across the genome (Segovia-Ramírez et al. 2023). Therefore, our findings should be interpreted with caution, as definitive conclusions cannot be drawn without broader genomic data and integrative analyses. Nevertheless, this study represents an initial step toward understanding how mitochondrial genetic diversity may be maintained and structured within this species-rich genus.

## Supplementary Material

zoaf075_Supplementary_Data

## Data Availability

https://data.mendeley.com/datasets/6r76dfh4wv/4.
