# Supplementary material for: Exploring mitochondrial diversity in the species-rich genus Liolaemus: the interplay between isolation by distance and environmental thermal variability
Source: Curr Zool. 2025 Dec 16;72(5):639–56. doi: 10.1093/cz/zoaf075 (PMC13690584; doi:10.1093/cz/zoaf075)
Supplement: Supplementary file 1 [file zoaf075_supplementary_data.zip › Supplementary_Data/S6-Correlative matrix.docx]

**Supplementary S6: Correlation among different variables**

**Fig. 1.S6**: Correlation matrix among different variables for *Cytb* gene. Green rectangles indicate most correlated variables.

**Fig. 2.S6** Correlation matrix among six coefficient of variation (CV) for *Cytb* gene. Green rectangles indicate most correlated variables.

**Fig. 3.S6** Correlation matrix among different variables for *12S rRNA* gene. Green rectangles indicate most correlated variables.

**Fig. 4.S6** Correlation matrix among six coefficient of variation (CV) for *12S rRNA* gene. Green rectangles indicate most correlated variables.
