# Supplementary material for: Exploring mitochondrial diversity in the species-rich genus Liolaemus: the interplay between isolation by distance and environmental thermal variability
Source: Curr Zool. 2025 Dec 16;72(5):639–56. doi: 10.1093/cz/zoaf075 (PMC13690584; doi:10.1093/cz/zoaf075)
Supplement: Supplementary file 1 [file zoaf075_supplementary_data.zip › Supplementary_Data/S7-Evolutionary analyses.docx]

**Supplementary S7: Evolutionary approach: Ancestral states reconstruction, phylogenetic signal and evolutionary model tests**

I- For isolation by distance (IBD) slope (𝛽) and nucleotidic diversity (π) values, considering *Cytb* and *12S rRNA* genes, we visualized ancestral states reconstruction using “contMap” function (*Phytools* package; Revell, 2024). II- We tested for phylogenetic signal using Blomberg´s *K* parameter. For this we using “phylosig” function (*Phytools* package; Revell, 2024). Phylogenetic signal is a measure of the tendency of related species to resemble one another and Blomberg's *K* is a quantitative measure of this pattern (Blomberg et al., 2003). Those *K* values lower than 1 and closer to 0 indicate little or no phylogenetic signal associated to a random or convergent evolution of trait. On the other hand, those *K* values that are closer, equal or greater than 1, suggest stronger similarities among closely related species and thus indicate a substantial degree of trait conservatism (Blomberg et al., 2003). Finally, III- we explored three different evolutionary model tests employing “fitContinuous” function (*geiger* package; Harmon et al., 2008) and *ape* package (Paradis and Schliep, 2019). We test for: 1-Brownian Motion evolutionary model (BM), where the evolutionary change in a trait occurs as result from random fluctuations through time (Felsenstein, 1988; Harmon et al., 2010). 2-Ornstein–Uhlenbeck model (OU), where the trait varies in relation to one or more evolutionary optima, at least in part of the lineage (Hansen, 1997; Butler and King, 2004) and finally 3- Early Burst (EB), in the case that a trait changes early in the evolutionary tree and then the rate of evolution slows down or fasts up gradually. We used Akaike's criterion corrected for small sample size (AIC_C_) to select the best fit models (Appendix S2), which has the lowest value for AIC_C_ and the highest Akaike's weight (AIC_Cwi_; Burnham and Anderson, 2004).

**Results**

**Fig. 1 S7:** Ancestral states reconstruction considering (A-B) IBD β and (C-D) nucleotide diversity (π) for *Cytb* and *12S rRNA* genes. In A, B, C can be observed higher values in separated phylogenetic species, while in D a higher value in phylogenetic closely related species.

**Table 1 S7:** Blomberg's *K* values (*K*) for phylogenetic signal patterns and their corresponding *P* statistical values. In bold those values statistically significant (*P* < 0.05).

|  | *K* | *P* |
| --- | --- | --- |
| β-*Cytb* | 0.052 | 0.06 |
| **π-*Cytb*** | **0.054** | **0.014** |
| β-*12S rRNA* | 0.09 | 0.38 |
| **π-*12S rRNA*** | **0.409** | **0.001** |

**Table 2 S7:** Evolutionary model parameters for IBD slope (β) and nucleotide diversity (π) for both genes, *Cytb* and *12S rRNA* considering Brownian Motion (BM), Ornstein–Uhlenbeck (OU) and Early Burst (EB) models. In bold those better models based on Akaike's criterion corrected for small sample size (AIC_C_) (lowest value of AIC_C_ and the highest Akaike's weight —AIC_CWi_).

|  | AICcBM | AICcOU | AICcEB | AICcwiBM | AICcwiOU | AICcwiEB |
| --- | --- | --- | --- | --- | --- | --- |
| β-*Cytb* | -561.666 | **-609.5172** | -559.492 | 4.06e^-11^ | **0.99** | 1.37e^-11^ |
| **π-*Cytb*** | -395.267 | **-443.1147** | -393.121 | 4.07e^-11^ | **0.99** | 1.39e^-11^ |
| β-*12S rRNA* | -292.216 | **-301.8922** | -289.7890 | 0.007 | **0.98** | 0.002 |
| **π-*12S rRNA*** | **-109.3770** | **-109.8237** | -107.0027 | **0.39** | **0.49** | 0.12 |

**References:**

Blomberg SP, Garland JRT, Ives AR, 2003. Testing for phylogenetic signal in comparative data: Behavioral traits are more labile. *Evolution* **57**: 717–745.

Burnham KP, Anderson DR, 2002. *Model selection and multimodel inference: A practical information-theoretical approach (2nd ed.).* Springer New York, NY.

Butler MA, King AA, 2004. Phylogenetic comparative analysis: a modeling approach for adaptive evolution. *AmerNat*, **164**(6), 683–695.

Felsenstein J, 1988. Phylogenies and quantitative characters. *Ann Rev Ecol Syst*, 445–471.

Hansen TF, 1997. Stabilizing selection and the comparative analysis of adaptation. *Evolution*, **51**(5), 1341–1351.

Harmon LJ, Weir JT, Brock CD, Glor RE, Challenger W, 2008. GEIGER: investigating evolutionary radiations. *Bioinformatics* **24**: 129–131.

Harmon LJ, Losos JB, Jonathan Davies T, Gillespie RG, Gittleman JL, et al., 2010. Early bursts of body size and shape evolution are rare in comparative data. *Evolution* **64**: 2385–2396

Paradis E, Schliep K, 2019. ape 5.0: an environment for modern phylogenetics and evolutionary analyses in R. *Bioinformatics* **35**: 526–528.

Revell LJ, 2024. phytools 2.0: an updated R ecosystem for phylogenetic comparative methods (and other things). *PeerJ* **12**: e16505.
