# Supplementary material for: Exploring mitochondrial diversity in the species-rich genus Liolaemus: the interplay between isolation by distance and environmental thermal variability
Source: Curr Zool. 2025 Dec 16;72(5):639–56. doi: 10.1093/cz/zoaf075 (PMC13690584; doi:10.1093/cz/zoaf075)
Supplement: Supplementary file 1 [file zoaf075_supplementary_data.zip › Supplementary_Data/S9.pdf]

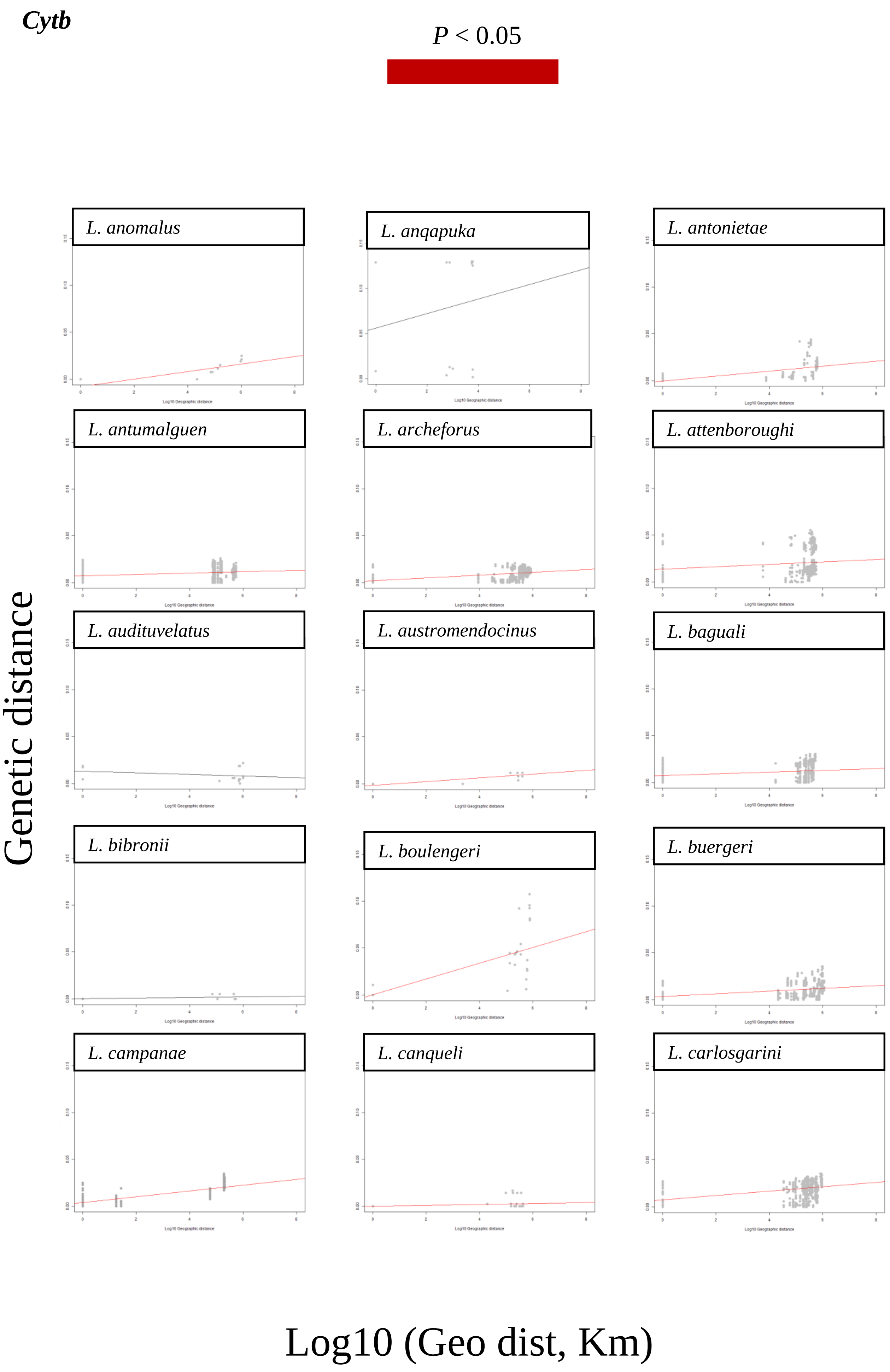
Cytb
P < 0.05
L. anomalus
L. anqapuka
L. antonietae
L. antumalguen
L. archeforus
L. attenboroughi
L. audituvelatus
L. austromendocinus
L. baguali
L. bibronii
L. boulengeri
L. buergeri
L. campanae
L. canqueli
L. carlosgarini
Genetic distance
Log10 (Geo dist, Km)

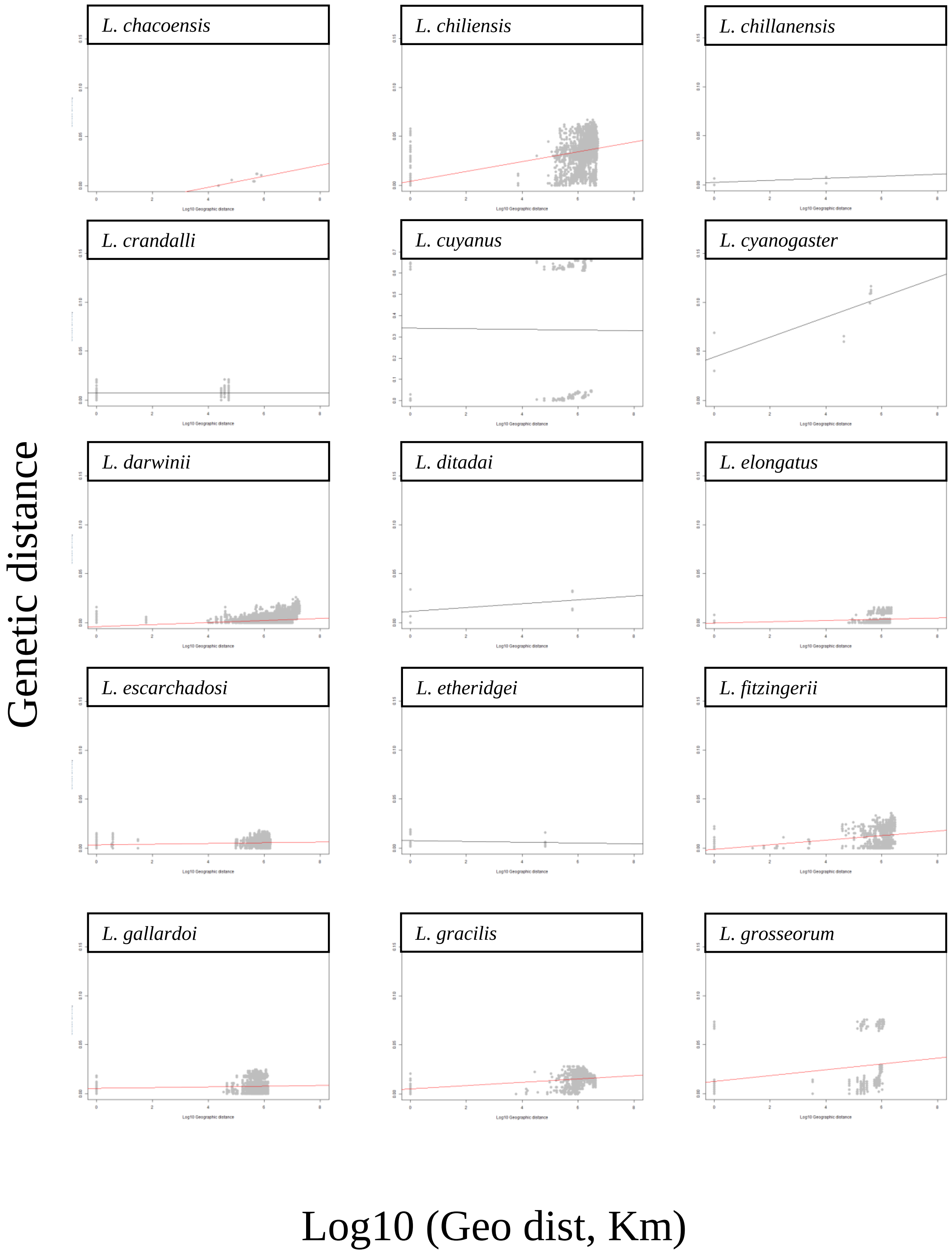
L. chacoensis
L. chiliensis
L. chillanensis
L. crandalli
L. cuyanus
L. cyanogaster
L. darwinii
L. ditadai
L. elongatus
L. escarchadosi
L. etheridgei
L. fitzingerii
L. gallardoi
L. gracilis
L. grosseorum
Genetic distance
Log10 (Geo dist, Km)

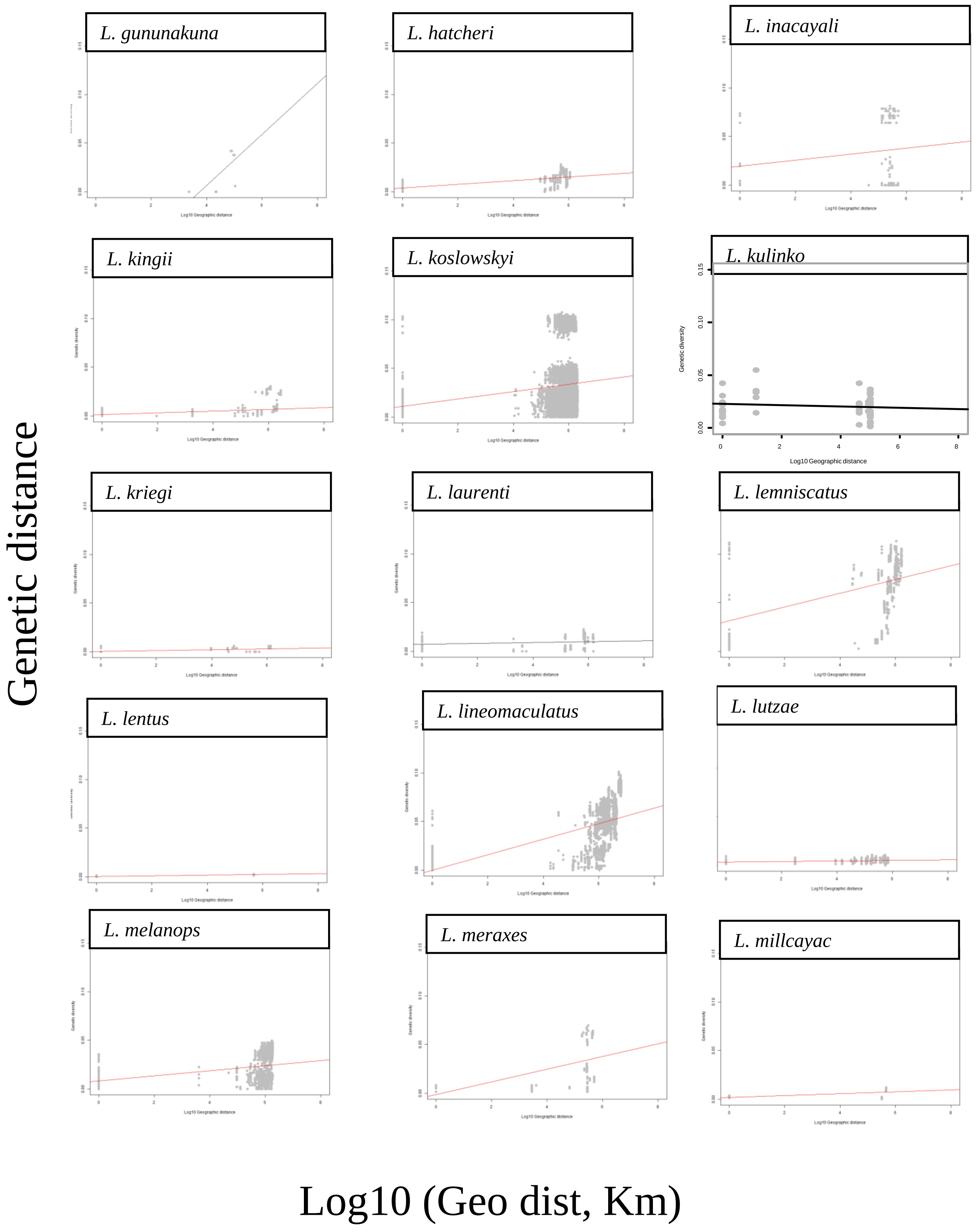

Genetic distance
L. gununakuna
L. hatcheri
L. inacayali
L. kingii
L. koslowskyi
L. kulinko
Genetic diversity
Log10 Geographic distance
L. kriegi
L. laurenti
L. lemniscatus
L. lentus
L. lineomaculatus
L. lutzae
L. melanops
L. meraxes
L. millcayac
Log10 (Geo dist, Km)

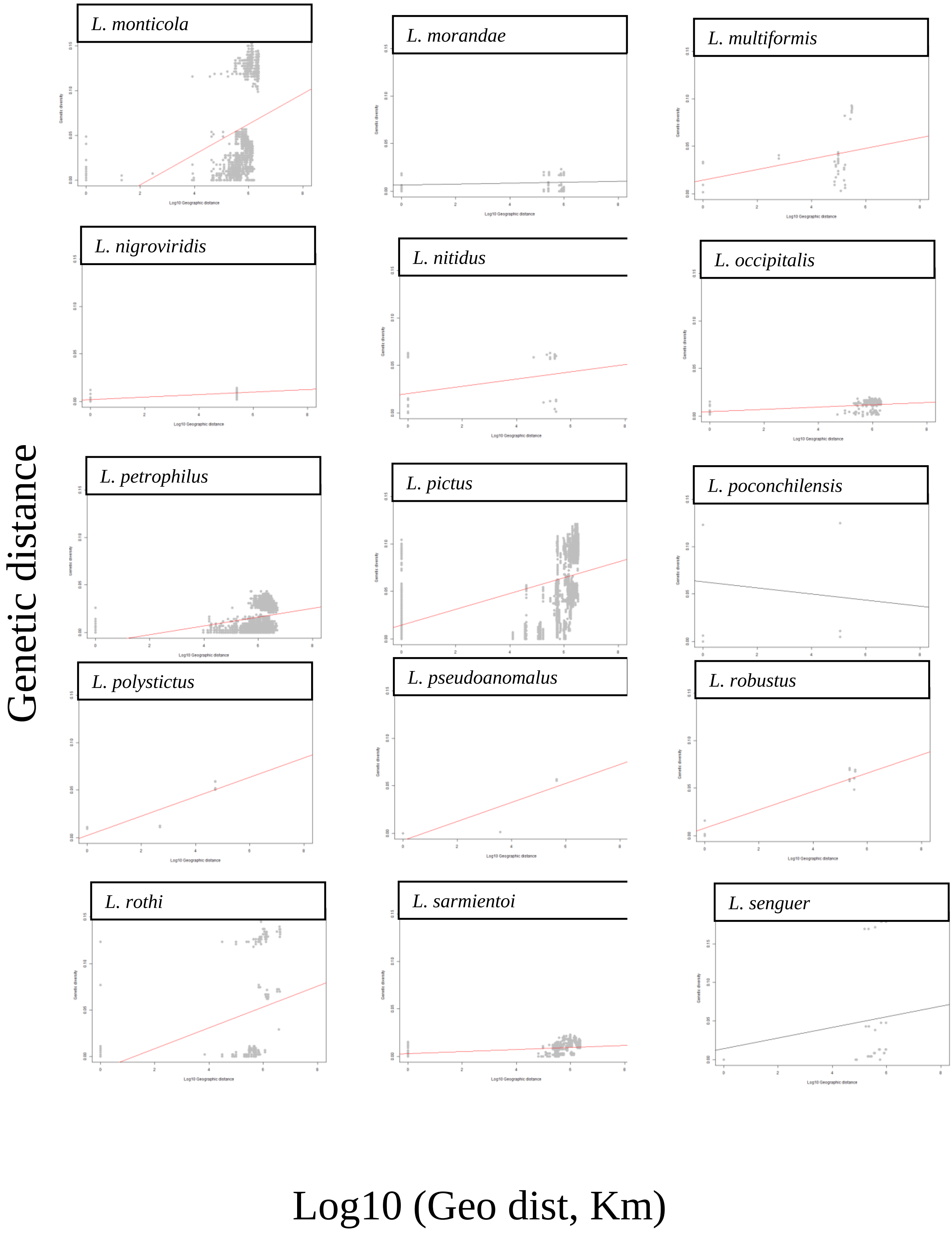
L. monticola
L. morandae
L. multiformis
L. nigroviridis
L. nitidus
L. occipitalis
L. petrophilus
L. pictus
L. poconchilensis
L. polystictus
L. pseudoanomalus
L. robustus
L. rothi
L. sarmientoi
L. senguer
Genetic distance
Log10 (Geo dist, Km)

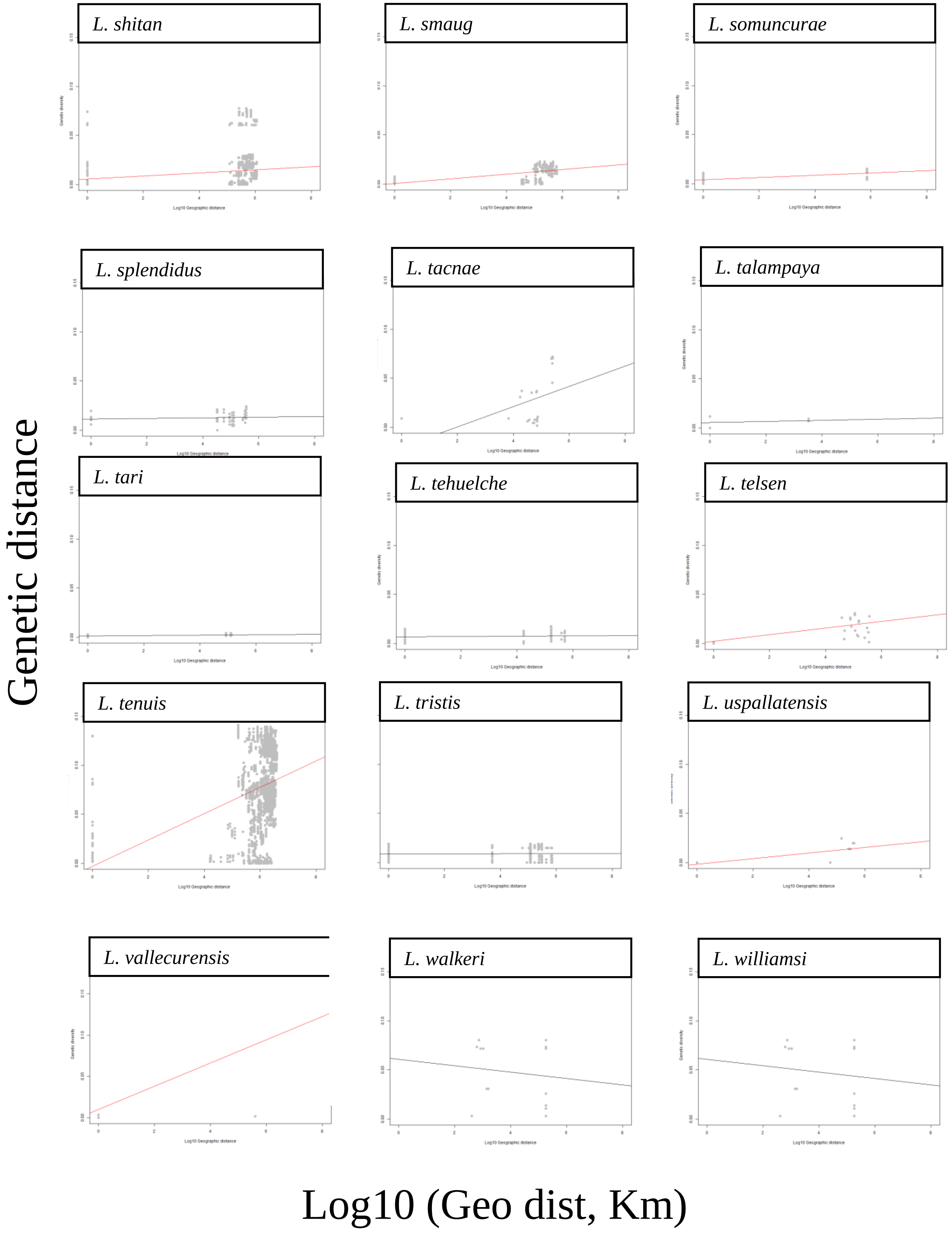
Genetic distance
L. shitan
L. smaug
L. somuncurae
L. splendidus
L. tacnae
L. talampaya
L. tari
L. tehuelche
L. telsen
L. tenuis
L. tristis
L. uspallatensis
L. vallecurensis
L. walkeri
L. williamsi
Log10 (Geo dist, Km)

12S rRNA

$P < 0.05$

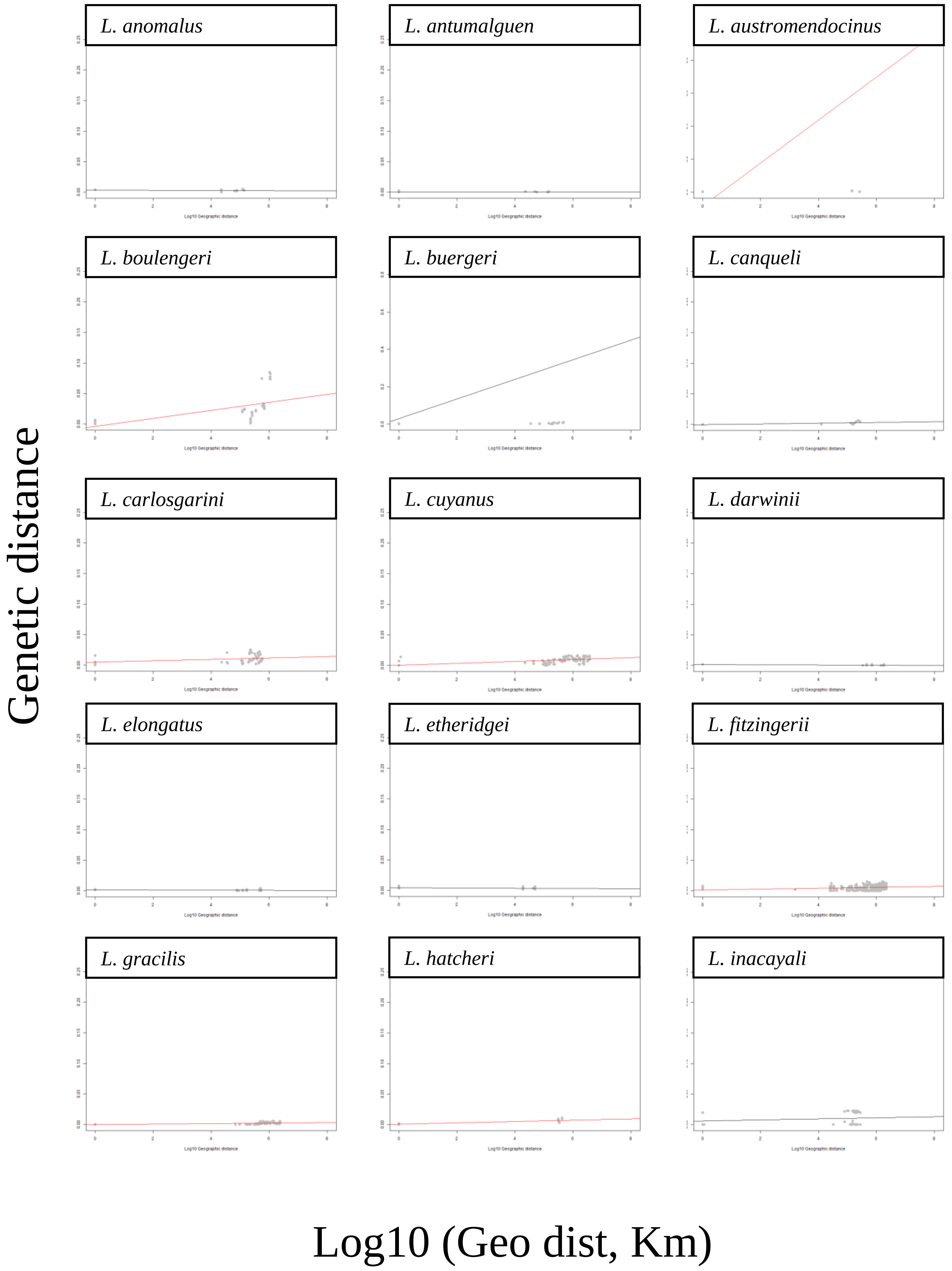

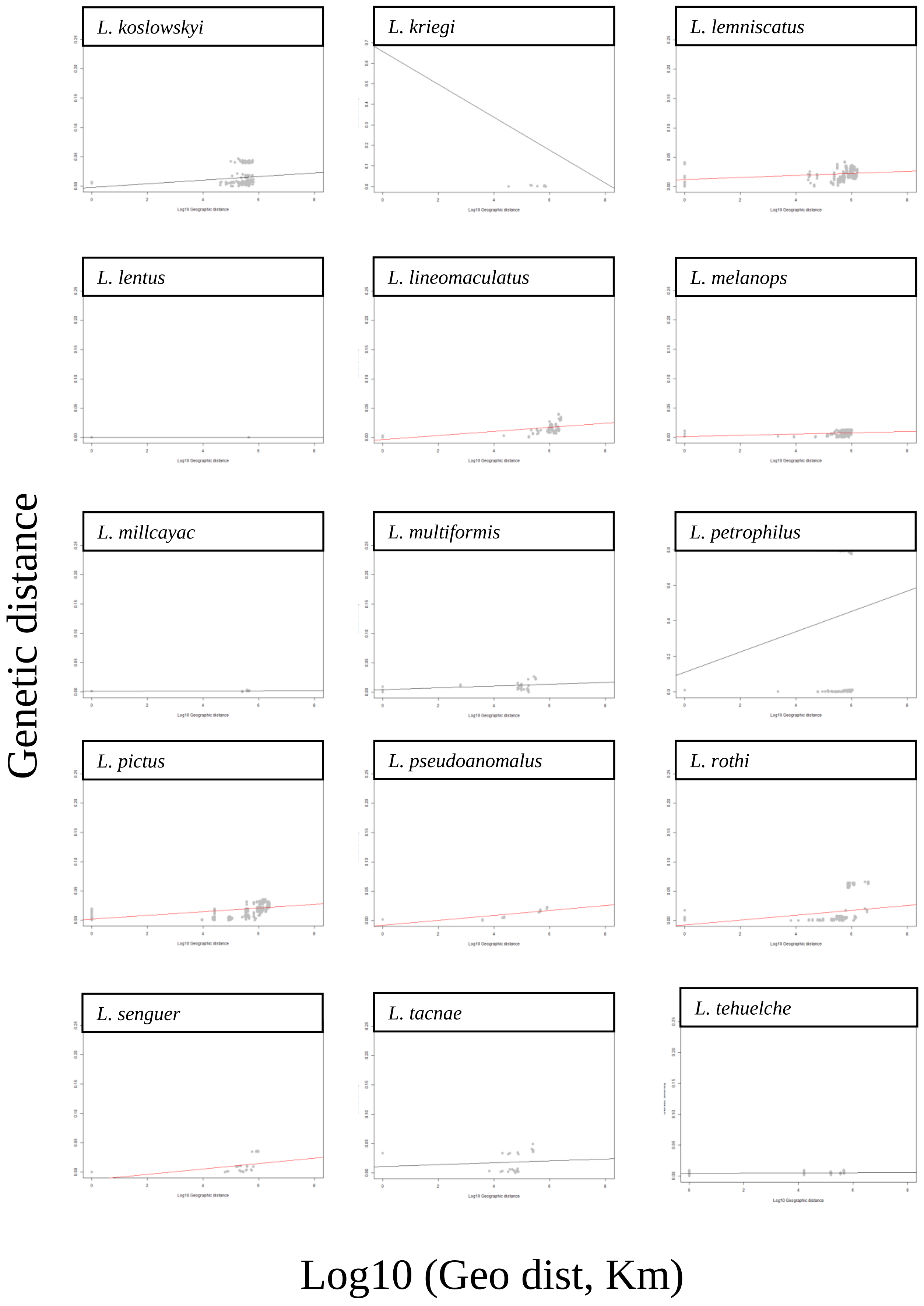

L. koslowskyi
L. kriegi
L. lemniscatus
L. lentus
L. lineomaculatus
L. melanops
L. millcayac
L. multiformis
L. petrophilus
L. pictus
L. pseudoanomalus
L. rothi
L. senguer
L. tacnae
L. tehuelche
Genetic distance
Log10 (Geo dist, Km)

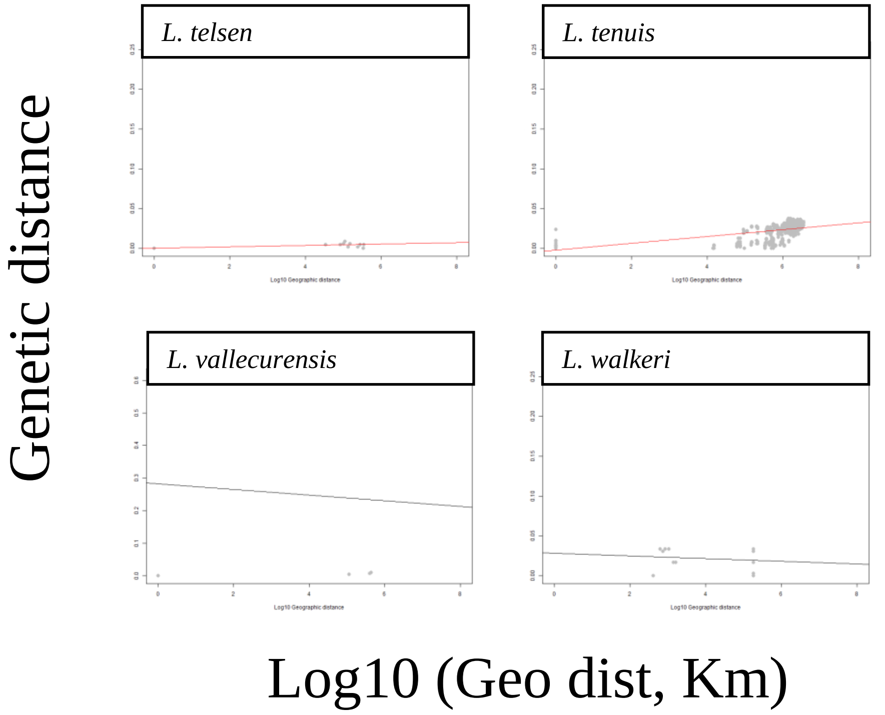
L. telsen
L. tenuis
L. vallecurensis
L. walkeri
Genetic distance
Log10 (Geo dist, Km)
